# Development, Usability, and Feasibility of a Pain-Stratified Smartphone-Based Home Intervention System for Patients With Adhesive Capsulitis: Exploratory Feasibility and Usability Trial

**DOI:** 10.2196/107314

**Published:** 2026-11-05

**Authors:** Hsun-Han Hsu, Kai-Chia Cheng, Ya-Lan Chiu

**Affiliations:** 1 Department of Physical Therapy and Assistive Technology, National Yang Ming Chiao Tung University, Taipei, Taiwan; 2 Glow Physical Therapy Center, Kaohsiung, Taiwan; 3 National Center for Geriatrics and Welfare Research, National Health Research Institutes, Yunlin, Taiwan; 4 Department of Physical Therapy, College of Medicine, National Cheng Kung University, No.1, University Road, Tainan, 701, Taiwan, 886 62353535 ext 5935; 5 Physical Therapy Center, National Cheng Kung University Hospital, Tainan, Taiwan

**Keywords:** frozen shoulder, adhesive capsulitis, telerehabilitation, mobile health, mHealth, smartphone app, home exercise, exercise adherence, range of motion

## Abstract

**Background:**

Frozen shoulder causes prolonged pain and restricted range of motion that impair daily function. Home exercise is an indispensable part of self-management, but adherence is often suboptimal under traditional printed or verbal instruction because of the lack of objective monitoring and individualized progression. Existing rehabilitation apps offer sensor-based assessment and feedback but generally lack an adaptive mechanism that tailors the exercise prescription to a patient’s dynamically changing clinical status.

**Objective:**

This study aimed to develop a personalized, pain-stratified telerehabilitation app for patients with frozen shoulder; to evaluate its usability, feasibility, and safety as an add-on to usual care; and to explore the system’s clinical outcomes on a descriptive basis.

**Methods:**

An Android app integrating smartphone sensor-based range of motion self-assessment, pain-based exercise stratification, usage records, and exercise reminders was developed. In an exploratory, assessor-blinded, randomized controlled feasibility trial, 10 patients with frozen shoulder were randomly allocated to an app group (n=5) or a handout group (n=5), and both underwent a 4-week home exercise intervention with identical content. Primary outcomes included system usability (System Usability Scale) and feasibility (retention and exercise adherence). Secondary outcomes focused on the exploratory clinical signs included pain and function (Shoulder Pain and Disability Index) and range of motion (measured by goniometer). Given the small sample size, continuous variables were analyzed nonparametrically and interpreted strictly as descriptive clinical trends without inferential hypothesis testing.

**Results:**

All 10 participants completed the trial (attrition rate 0%). The mean System Usability Scale score in the app group was 87.5 (SD 5.86), corresponding to an “excellent” usability rating. Over the 28-day intervention, the app’s usage logs objectively documented a mean of 18.0 (SD 5.0) of 28 exercise days (18/28, 64.3%) in the app group, demonstrating the feasibility of objective home monitoring. Both groups improved in Shoulder Pain and Disability Index scores and range of motion. Notably, all participants in the app group achieved a descriptive within-group improvement in the clinically challenging shoulder external rotation, whereas the handout group showed smaller marginal gains; however, between-group comparisons across all parameters were interpreted descriptively without significance testing due to the sample size. On the other clinical outcomes, the handout group showed numerically comparable or larger improvements.

**Conclusions:**

This study shows that a pain-stratified, personalized telerehabilitation app is highly usable and feasible as an add-on mobile health intervention for patients with frozen shoulder and that it provides objective home monitoring of exercise adherence that traditional handouts cannot. These exploratory findings highlight the need for a fully powered confirmatory trial. To robustly evaluate clinical efficacy, future study designs must include a common objective adherence measure, stratification variables, and an extended follow-up period.

## Introduction

Frozen shoulder, also clinically termed adhesive capsulitis, is pathologically characterized by excessive scar tissue formation and fibrotic adhesions within the shoulder joint capsule, leading to severe pain, joint stiffness, and progressive functional impairment of the shoulder [1]. The literature reports that the prevalence of this condition is approximately 3% to 5% in the general population, with a particular predilection for middle-aged and older women aged 40 to 60 years [2]. Although the precise pathogenesis remains incompletely understood, established clinical risk factors include female sex, diabetes mellitus, and thyroid disease [3-5].

Clinically, frozen shoulder presents primarily with spontaneous pain and global restriction of shoulder range of motion, most notably in the planes of flexion, abduction, internal rotation, and external rotation [6-8]. The condition not only impairs shoulder function but also leads to reduced quality of life, work disability, and sleep disturbance [9], imposing a substantial socioeconomic burden due to direct medical costs and work absence [10]. While clinical treatment strategies are diverse [1-3], early professional medical intervention is strongly recommended to substantially improve prognosis and shorten the disease course [2,3,11]. According to the clinical practice guidelines issued by the American Physical Therapy Association (APTA) [3], patient education and home exercise training play an indispensable role in overall self-management, with specific program designs, such as incorporating scapulothoracic strengthening, showing marked benefits [12]. However, previous studies strongly caution that overly aggressive physical interventions can overstimulate the inflamed joint capsule, leading to secondary injury [13,14]. Conversely, performing self-directed exercises within a pain-free range achieves significantly better long-term functional outcomes [13]. Therefore, in line with APTA guidelines, exercise prescriptions must be strictly stratified according to the patient’s tissue irritability level [3]. For patients with high irritability, treatment should focus on active-assisted mobility exercises within a pain-free range rather than high-intensity tissue stretching, underscoring the necessity of personalized training designs that allow patients to dynamically adjust their routine according to their symptoms.

Nevertheless, although a well-structured and personalized home exercise design is critical to the prognosis of frozen shoulder, the practical realization of these clinical benefits remains highly dependent on patient exercise adherence in an unsupervised environment [15,16]. When patients perceive treatment as ineffective or experience a sense of helplessness, exercise discontinuation frequently ensues [17]. These challenges highlight 3 major gaps in traditional home programs that rely heavily on printed or verbal instructions. First, health care providers are unable to monitor patient adherence in real time [16,17]. Second, patients often forget movement details in the home environment; without immediate professional feedback, they risk secondary injury caused by improper compensation [16]. Third, the lack of a dynamic monitoring mechanism prevents therapists from tracking daily changes in pain and joint angles, making it difficult to fine-tune prescriptions in a timely manner [18].

With the rapid advancement of mobile health (mHealth) and digital health technologies, smartphone apps have become an innovative solution to overcome the spatial and temporal constraints of traditional health care [19,20]. These systems can automatically record usage, using built-in sensors or image-based methods to measure shoulder joint angles and provide real-time visual feedback [21]. Numerous scholars have developed frozen shoulder apps equipped with range of motion measurement and audio-visual guidance [22,23]; however, these systems mostly provide one-size-fits-all static prescriptions, lacking the clinical decision-making logic for personalized, stratified adjustments based on the patient’s dynamic pain status [22-25]. This disconnect between self-assessment and exercise prescription renders them unable to adapt to the dynamically changing tissue irritability of patients across different stages, limiting the substantive clinical benefits of digital telerehabilitation. Crucially, neither traditional printed handouts nor reminder-only apps can dynamically adjust exercise intensity in response to a patient’s fluctuating tissue irritability. Furthermore, while existing sensor-equipped apps provide basic range of motion tracking, they typically deliver static exercise prescriptions decoupled from the day-to-day pain variations that clinical guidelines use to grade rehabilitation intensity. Therefore, the present system was specifically designed to bridge this clinical gap by closing the “self-assessment-to-prescription” loop in an unsupervised home setting.

Accordingly, the objectives of this study were 3-fold. The primary aim was to develop a personalized, pain-stratified smartphone intervention system capable of dynamically tailoring exercise prescriptions to the patient’s real-time pain status. The second aim was to evaluate the system’s usability and feasibility as an add-on to usual care. Finally, the third aim was to explore its clinical usefulness and generate essential variance parameters to inform the design of future, adequately powered confirmatory trials.

## Methods

### App Development

#### Development Environment and App Functions

This study selected Google’s Android operating system as the platform for smartphone apps because Android is the most widely used smartphone operating system in Taiwan. The software development tool used in this study was Android Studio, with Java and XML as the programming languages. The app comprised 5 main functions: frozen shoulder educational content, self-assessment, personalized exercise, usage records, and an exercise reminder. Screenshots of the app are shown in Figure 1.

**Figure 1.**
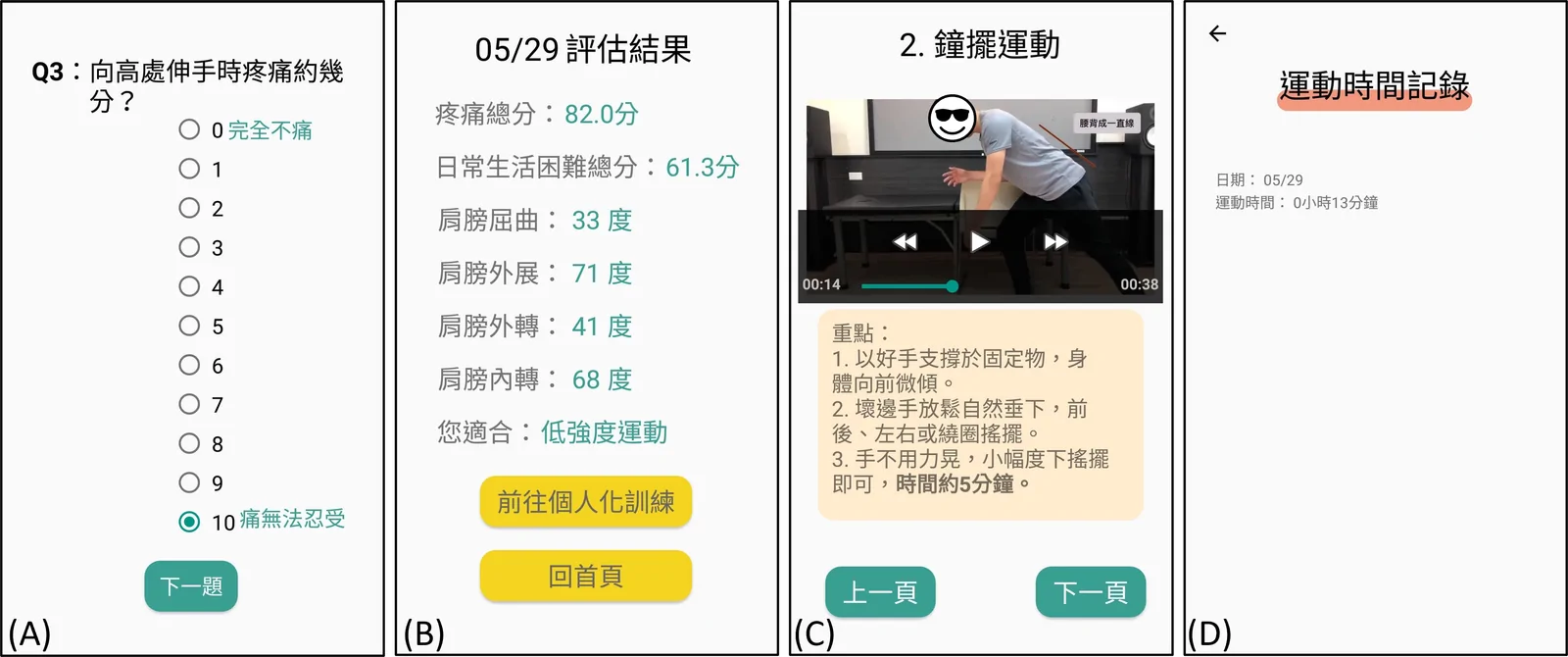
Screenshots of the Android frozen shoulder telerehabilitation app: (A) Shoulder Pain and Disability Index (SPADI)–based pain self-assessment, (B) daily self-assessment result with the system-recommended exercise intensity, (C) video-based exercise instruction, and (D) exercise-time record.

#### Frozen Shoulder Educational Content

According to recommendations from previous studies, enhancing the educational knowledge of patients with frozen shoulder can help them manage their symptoms and reduce fear [3,14]. By informing patients of the causes of frozen shoulder and symptoms that change over time, patients can gain advance understanding of the symptom trajectory, and further recommendations can be made regarding exercises appropriate for different stages, along with relevant precautions. The educational content on frozen shoulder incorporated in this app includes an introduction to frozen shoulder, disease staging, and treatment options, enabling patients to better understand their own condition through this educational content and to ease their fear of the symptoms.

#### Self-Assessment

The assessment items included pain, difficulty with activities of daily living, and range of motion. The personalized exercise program designed in this study used the pain level to determine the intensity of the personalized exercise subsequently recommended by the system.

Assessments of pain and difficulty in activities of daily living were adapted from the Shoulder Pain and Disability Index (SPADI), chosen for its established validity and responsiveness [26]. Administered as a digital questionnaire, it evaluated the affected shoulder’s status over the past week through 2 sections: a 5-item pain subscale (rated from 0, “no pain at all,” to 10, “unbearable pain”) and an 8-item functional difficulty subscale (rated from 0, “no difficulty at all,” to 10, “complete inability”). To ensure data completeness, the app required users to provide an answer for each item sequentially before proceeding to the next.

The range of motion assessment comprised 4 shoulder movements: shoulder flexion, shoulder abduction, shoulder external rotation, and shoulder internal rotation. Movement descriptions and demonstration videos were provided for users to view and review. The videos included precautions for each movement—such as the starting position during measurement and avoiding shoulder shrugging—prompting users to complete the assessment in the correct posture. The smartphone was secured to the user’s wrist with an arm strap, allowing the sensor to detect the 3-axis rotation angles of the phone in accordance with the movement as a measure of shoulder range of motion.

After the user tapped Start, the first movement began. There was a 3-second preparation period, and upon hearing the start tone, the user began the movement and was required to reach the maximum extent of that movement (full range) within 5 seconds; the movement stopped upon hearing the stop tone, at which point the app displayed the change in range of motion on the screen in real time. Shoulder range of motion was calculated using the smartphone’s built-in rotation vector sensor: a built-in function was used to convert the sensor’s quaternion data into a rotation matrix, and a further function converted the rotation matrix into the smartphone’s rotation angles about the *x*-, *y*-, and *z*-axes. Azimuth is the angle of rotation of the phone about the *z*-axis, pitch is the angle of rotation about the *x*-axis, and roll is the angle of rotation about the *y*-axis. In this study, the smartphone’s rotation angle about the *z*-axis was used as the measurement value for shoulder flexion and abduction, and its rotation angle about the *x*-axis was used as the measurement value for shoulder external and internal rotation.

As for the dedicated app developed in this study, its reliability and validity relative to a conventional universal goniometer were evaluated by our research team in a separate technical validation study. Specifically, the smartphone app demonstrated excellent within-day intrarater reliability across all movements (intraclass correlation coefficients >0.95; SEM 2.5°-5.0°) and adequate between-day intrarater reliability (intraclass correlation coefficients ranging from 0.83 to 0.95). Furthermore, criterion-related validity showed a highly significant correlation with the universal goniometer (Pearson correlation coefficients >0.91) across all 4 shoulder motions. The concordance correlation coefficients showed high concordance for flexion (0.98), abduction (0.95), and internal rotation (0.92), with slightly lower concordance for external rotation (0.84), which is appropriately attributed to possible trunk compensatory movements. Given this established technical foundation, the present trial focuses primarily on clinical feasibility and user experience following system intervention. The reliability and validity of these sensor-based measurements were evaluated in a separate study; the corresponding indices are provided in Multimedia Appendix 1.

After all 4 ranges of motion were measured, the final page presented the user’s assessment results for that day, including the recommended exercise intensity. The assessment results were automatically saved within the usage records function.

#### Personalized Exercise Training

According to the 2013 APTA guidelines [3], physical therapists should determine appropriate exercise content on the basis of the patient’s overall irritability level. However, considering that this system is a telerehabilitation home-based intervention without real-time supervision by health care personnel, it is difficult for the smartphone sensor to fully exclude trunk compensation caused by patient pain when measuring range of motion. In addition, verification of this system’s sensor accuracy and consistency is currently under peer review (Y-L Chiu, PhD, PT, unpublished data, October 2026). To ensure absolute clinical safety of home-based rehabilitation and to prevent the system from prescribing an excessively high exercise intensity based on erroneous range of motion values measured under compensation, the system’s personalization algorithm currently adopts a conservative strategy, using the patient’s subjective pain level as the sole decision parameter for progression of prescription intensity. Pain was captured with the 5-item SPADI pain subscale, each item rated from 0 (no pain at all) to 10 (unbearable pain), and the mean of the 5 items—yielding a single 0 to 10 pain score—was used as the stratification indicator. On the basis of this mean pain score, the app classified the recommended exercise intensity into 3 levels: low intensity for severe pain (mean pain score ≥7), medium intensity for moderate pain (4≤ mean pain score ≤6), and high intensity for mild pain (mean pain score ≤3). For the clinical outcome analysis and on the app’s results screen (Figure 1B), the same subscale is expressed as a 0 to 100 percentage; the 0 to 10 mean and the 0 to 100 percentage are equivalent, differing only by a factor of 10.

The exercise content was designed with reference to previous literature and included active-assisted exercises, stretching exercises, muscle strengthening exercises, and correct-posture reminders, with each intensity level containing different exercise content [3,13,27]. For the user interface design presenting the exercise content, reference was made to the presentation approach of previous similar apps; to prevent users from forgetting the exercise movements and to allow them to view the content at any time, the exercise content in this app was presented in video form with accompanying key reminders [21-23]. The detailed exercise list is provided in Multimedia Appendix 2.

#### Usage Records and Exercise Reminder

The record-keeping function was implemented using SQLite, the database built into Android Studio, and contained both assessment results and exercise time data. The app was designed with an alarm reminder to encourage users to exercise regularly each day. Using sound and vibration as the notification method, notifications popped up at 9:00 AM and 9:00 PM each day.

### Intervention Trial

#### Study Design

This study used an exploratory, assessor-blinded, randomized controlled feasibility trial. To comprehensively evaluate the benefits of the digital health intervention, the study design was organized around 2 main axes: first, examining the system usability and feasibility of the smartphone app as a home-based rehabilitation tool, and second, using random allocation to divide participants into an app group and a handout group to undergo 4 weeks of home exercise intervention with identical content, in order to evaluate exercise adherence as a core feasibility metric, while assessing pain levels and shoulder range of motion strictly as exploratory secondary outcomes rather than to establish comparative clinical efficacy. Participant recruitment was conducted from March to June 2019, and the subsequent intervention and follow-up period extended to July 2019. Participants were recruited from rehabilitation clinics and communities in Beitou District, Taipei City, and were screened to meet the inclusion criteria.

#### Participants

The inclusion criteria were shoulder pain symptoms for at least 4 months, restricted shoulder range of motion (shoulder external rotation <45° or restricted movement in 2 or more planes), and the ability to operate a smartphone. Exclusion criteria included normal passive range of motion, glenohumeral osteoarthritis, neuropathic pain, and corticosteroid injection within the preceding 3 months [23]. A total of 10 participants (n=5 per group) completed the evaluation. While unpowered for clinical efficacy, this sample size aligns with methodological guidelines for designing and evaluating early-stage feasibility and usability trials [28,29]. A pragmatic cohort of 10 to 12 participants is generally considered sufficient to identify primary usability issues, evaluate user acceptability, and assess core feasibility parameters prior to a larger trial [30]. Consequently, the clinical component of this study is strictly intended for exploring clinical usefulness (exploratory) and generating variance estimates to inform an adequately powered future definitive trial, rather than to conduct inferential hypothesis testing or establish comparative efficacy.

#### Study Instruments

In the intervention trial, the app group installed the frozen shoulder app developed in this study; if a participant used a smartphone running a non-Android operating system, the researchers lent them a Zenfone 4 (ASUS) smartphone. In addition, the researchers provided participants in the app group with a smartphone arm strap to assist with self-assessment. Participants allocated to the handout group were given a copy of a frozen shoulder educational handout produced by the research team (Multimedia Appendices 3 and 4).

#### Study Procedure

After participants met the eligibility criteria and provided consent, the researchers first recorded their baseline demographic data and then allocated them in a 1:1 ratio to the app group or the handout group. Allocation used sealed, opaque, and outwardly identical lots (5 per group) that had been prepared and shuffled in advance by a researcher not involved in outcome assessment; each participant drew 1 lot at enrollment, and the identical appearance of the lots concealed the allocation until assignment. After group allocation, a pretest was conducted, comprising pain level, level of difficulty in activities of daily living, and shoulder range of motion. To ensure blinding, the pretests and posttests of both groups were administered by a physical therapist who was blinded to group allocation, covering the questionnaires and shoulder range of motion, with range of motion measured using a conventional goniometer. For the app group, in addition to the conventional measurement method, another researcher assisted in installing the app on the participant’s smartphone on the same day and, based on the pretest assessment results, assisted in confirming the training program recommended by the system for the first week’s exercise intensity, which was automatically generated according to the assessment results. The researchers instructed participants in the app group on how to use the app and taught those in the handout group the educational exercises.

The intervention lasted 4 weeks in total, and each participant was required to perform exercise training at least once daily; in addition, the app group was required to perform self-assessment at least once per week in order to continuously update their personalized training program. Each week, the researchers contacted participants via a messaging app to determine whether they were experiencing any difficulty operating the smartphone app or performing the exercises. In the handout group, the frequency and duration of that week’s exercise were recorded using 2 questions: “How many times did you perform the exercises this week?” and “About how long did each exercise session last?” Participants in the app group were instead asked to provide screenshots of the app’s built-in training logs. If a participant had questions, the researchers scheduled a time to discuss the issues in person. After 4 weeks, a posttest was conducted, with the same items and procedure as the pretest.

#### Intervention Content and Usual Care Co-Interventions

Both groups performed a home exercise program with identical content. The exercise intensity was divided into 3 levels (low, medium, and high); in the app group, the system automatically assigned the training difficulty according to the assessment results, whereas in the handout group, the difficulty was set by the researchers. All 3 levels included correct-posture reminders. Low-intensity training was to be performed at least once daily for at least 15 minutes per session, whereas the other 2 intensity levels were to be performed at least once daily for at least 20 minutes per session.

Importantly, during the 4-week intervention, participants in both groups continued to receive their routine medical care for frozen shoulder under the National Health Insurance system. This usual care included physical therapy modalities and pharmacological treatments. However, the specific types, dosages, and frequencies of these co-interventions were not quantitatively recorded or controlled for as covariates in this study.

### Ethical Considerations

The experimental protocol of this study was approved by the Institutional Review Board of National Yang-Ming University (approval number YM107116F). Written informed consent was obtained from all participants prior to enrollment. To maintain privacy and data confidentiality, all participant information was deidentified. Participants did not receive any compensation. This trial was not registered in a public registry. This is acknowledged as a limitation (see the *Limitations* section). No changes were made to the trial design, eligibility criteria, or study procedures after the trial commenced.

### Outcome Measures

#### Primary Outcome

The primary outcomes focused on trial feasibility. Indicators included participant retention rate and intervention safety (adverse event incidence). To collect exercise adherence data in a realistic clinical setting, researchers contacted both groups weekly. The handout group provided interview-based self-reports of their exercise frequency, whereas the app group was asked to provide screenshots of the app’s built-in training logs, rather than verbally report their exercise frequency. Because the 2 methods differ fundamentally, the resulting adherence values are not comparable between groups. Adherence was therefore summarized separately for each group as a descriptive feasibility metric, with the app group’s logs illustrating the app’s capacity for objective home monitoring. Each participant’s exercise adherence was tabulated after 4 weeks, calculated as follows:

Exercise adherence (%) = (total number of exercise days) / 28 days × 100

To evaluate the usability of the frozen shoulder app in this study, the System Usability Scale (SUS) was used, with a maximum total of 100 points, where higher scores indicate greater satisfaction with system usability [31]. In addition to the SUS scores, each participant in the app group provided brief open-ended feedback on their experience of using the app at the end of the intervention. These responses were collected and summarized to identify common views regarding the user experience.

#### Secondary Outcomes

Pain and difficulty in activities of daily living were assessed using the SPADI, which comprises 13 self-rated items—5 for the pain subscale and 8 for the activities-of-daily-living difficulty subscale. Each item was rated from 0 to 10, with 0 indicating no pain at all or no difficulty at all and 10 indicating unbearable pain or extreme difficulty. The pain and activities-of-daily-living difficulty subscales were scored separately, and each subscale score was converted to a percentage ranging from 0 to 100, calculated as follows:

Pain subscale score (%) = (sum of the 5 pain item scores) / 50 × 100

Activities-of-daily-living difficulty subscale score (%) = (sum of the 8 difficulty item scores) / 80 × 100

Active shoulder range of motion was assessed with a goniometer for 4 shoulder movements: shoulder flexion, shoulder abduction, shoulder external rotation with the arm at the side, and shoulder internal rotation. The goniometric measurement methods followed those published by Norkin and White [32] in 2016. Shoulder flexion and shoulder abduction were measured in the standing position, with the fulcrum at the acromion, measuring the angle between the vertical midline of the body and the midline of the upper arm; shoulder external rotation was measured in the standing position, with the starting position being the upper arm against the body and the elbow flexed at 90°, the fulcrum at the midpoint of the olecranon process of the ulna, measuring the change in angle of the midline of the ulna; shoulder internal rotation was measured in the supine position, with the fulcrum at the olecranon process, measuring the angle between the midline of the ulna and the vertical to the ground. Each movement was measured 3 times at full range, and the mean of the 3 measurements was calculated after recording. All outcome measures and their assessment procedures were defined before enrollment began, and no changes were made to the outcomes or their measurement methods after the trial commenced.

### Statistical Analysis

Consistent with the aims of a usability and feasibility study, feasibility indicators—recruitment, retention, adherence, system usability, and safety—were defined as the primary outcome domain and are summarized with descriptive statistics. Given the sample size (n=5 per group), clinical outcomes (pain, function, and range of motion) were treated as exploratory and are presented at the individual-participant level, with group means and SDs reported for context; no inferential hypothesis testing was performed on the clinical outcomes, and no *P* values are reported for these comparisons. Given the small sample size and in line with CONSORT (Consolidated Standards of Reporting Trials) recommendations, baseline characteristics are presented descriptively without significance testing.

## Results

### Participant Demographic Characteristics

Of the 20 individuals assessed for eligibility (recruited from rehabilitation outpatient clinics and a community activity center in the Beitou District of Taipei), 10 did not proceed to enrollment—because they did not meet the inclusion criteria (n=4) or declined to participate owing to time constraints (n=6)—and the remaining 10 patients with frozen shoulder were enrolled and randomly allocated to the app group (n=5) or the handout group (n=5); the participant flow is summarized in Figure 2 (CONSORT flow diagram). Group allocation was concealed until assignment: each participant drew 1 sealed, opaque, and identical lot at enrollment, yielding a 1:1 allocation (5 per group). All 10 participants completed the 4-week intervention, with no withdrawals (retention 100%). Baseline demographic characteristics, presented descriptively in Table 1, were broadly comparable between groups in age, sex, affected side, and symptom duration. One baseline difference is, however, relevant to the interpretation of the outcomes reported below: shoulder external rotation was considerably lower in the app group than in the handout group at entry (mean 20.6° vs 35.8°), leaving the app group with more room for improvement in that plane (Table 2).

**Figure 2.**
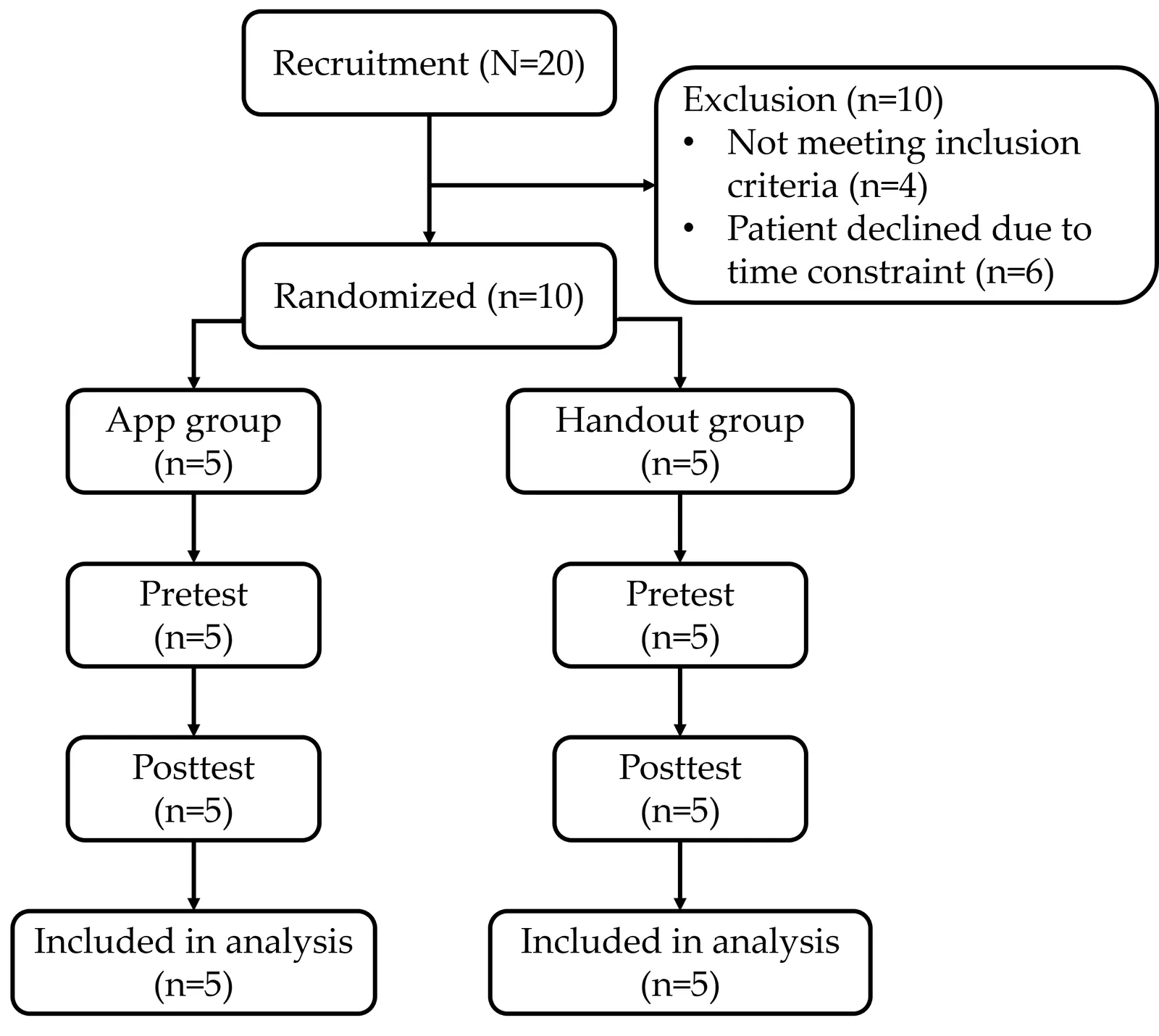
Participant flow (CONSORT [Consolidated Standards of Reporting Trials] diagram) for the assessor-blinded comparison of a smartphone-based home exercise system with an educational handout in adhesive capsulitis: screening, exclusions, 1:1 allocation, and completion (n=10).

**Table 1.** Baseline demographic and clinical characteristics of the 10 participants with adhesive capsulitis.

Characteristic	App group (n=5)	Handout group (n=5)
Age (y), mean (SD)	49.4 (5.0)	51.0 (9.4)
Female, n (%)	4 (80)	5 (100)
Affected side—right, n (%)	1 (20)	1 (20)
Symptom duration (mo), mean (SD)	7.67 (3.8)	7.40 (2.6)

**Table 2.** Clinical outcomes by group: pre-post values and mean change.

Outcome and group	Pre, mean (SD)	Post, mean (SD)	Change, mean
SPADI^a^ pain (%)			
App	44.4 (19.9)	30.0 (26.5)	−14.4
Handout	50.4 (13.6)	32.0 (28.0)	−18.4
SPADI function (%)			
App	40.5 (16.6)	26.2 (17.1)	−14.3
Handout	43.5 (19.8)	23.3 (21.9)	−20.2
Flexion (°)			
App	136.2 (28.7)	144.1 (20.8)	+7.9
Handout	142.3 (28.5)	153.3 (19.9)	+11.0
Abduction (°)			
App	121.8 (38.6)	128.1 (29.1)	+6.3
Handout	122.4 (35.6)	139.5 (28.6)	17.1
External rotation (°)			
App	20.6 (5.6)	30.5 (6.2)	+9.9
Handout	35.8 (21.8)	38.1 (19.6)	+2.3
Internal rotation (°)			
App	38.5 (10.7)	43.4 (7.2)	+4.9
Handout	47.5 (13.5)	51.5 (14.8)	+4.0

a SPADI: Shoulder Pain and Disability Index.

### Feasibility and Exercise Adherence

Feasibility, the primary outcome domain, was supported across all indicators. Retention was 100% (10/10) with no withdrawals, and no adverse events or exercise-induced pain exacerbations related to the home exercise program were reported in either group during the 4-week intervention. Over the 28-day intervention, exercise adherence was captured objectively through the app’s usage logs in the app group and through weekly interview-based self-reports in the handout group. Because these instruments differ fundamentally in how they capture behavior, the adherence values are reported descriptively as feasibility metrics and are not compared between groups. In the app group, the usage logs documented a mean of 18.0 (SD 5.0) exercise days (18/28, 64.3% adherence), illustrating the app’s capacity for continuous, objective home monitoring. The handout group’s self-reported values are shown in Table 3 for completeness only. The individual participants’ adherence is shown in Figure 3.

**Table 3.** Exercise adherence over the 4-week home intervention, reported separately for each group by data source^a^.

Group: data source	Adherence (d), mean (SD)	Completion rate (%)	Median (IQR)	Range	% meeting ≥3×/week
App group (n=5): app usage log^b^	18.0 (5.0)	64.3	21 (16-21)	10-22	80
Handout group (n=5): weekly self-report^c^	11.8 (3.9)	42.1	11 (9-13)	8-18	40

a Adherence was measured with different instruments in the 2 groups; values are presented descriptively and are not directly comparable between groups.

b Recorded in the app's built-in usage log and submitted weekly as screenshots.

c Reported by participants in weekly interviews (recall-based self-report).

**Figure 3.**
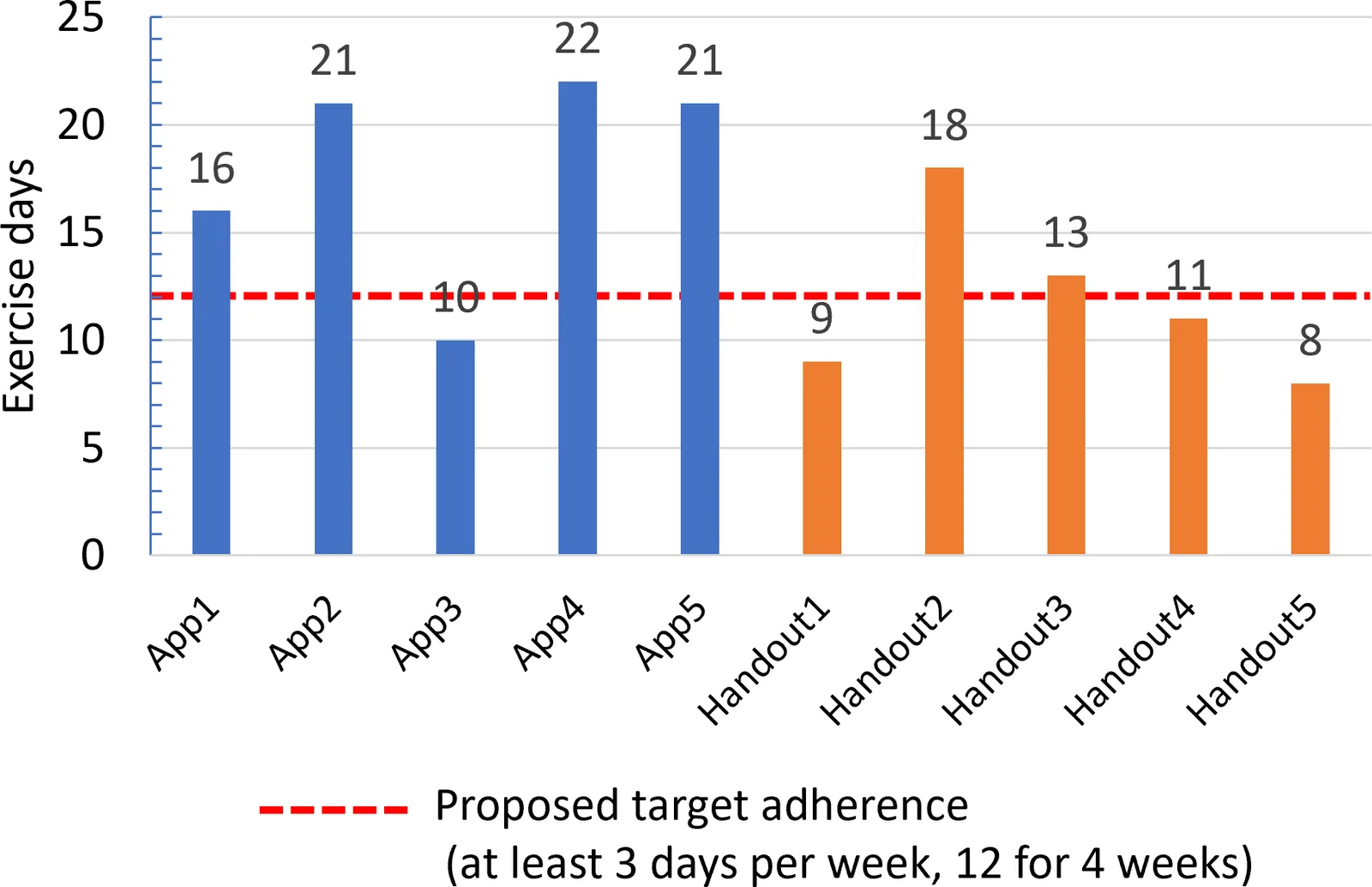
Individual participant exercise adherence over a 4-week home-based intervention for frozen shoulder, both app group and handout group. The red horizontal dashed line at 12 days indicates the proposed clinically reasonable target of completing home exercises at least 3 days per week. Adherence was derived from app usage logs in the app group and from weekly self-report in the handout group; values are not directly comparable between groups.

### System Usability

#### Overview

System usability was evaluated in the app group (n=5). The mean SUS score was 87.5 (SD 5.86), well above the established benchmark of 68 and corresponding to an “excellent” rating on the adjective rating scale of Bangor et al [31]. Individual scores ranged from 77.5 to 92.5; per-participant scores and open-ended feedback are presented in Table 4. Furthermore, qualitative review of the weekly follow-up communications revealed a complete absence of technical or operational troubleshooting requests. Specifically, participants reported no difficulties across the core practical domains of notifications, exercise performance, self-assessment execution, or daily usage recording. This lack of operational friction indicates that the initial face-to-face setup and tutorial effectively preempted potential technical barriers, further substantiating the system’s high practical feasibility and intuitive interface in a home setting. Recurring themes in the qualitative feedback were that the video demonstrations helped participants remember the exercises, the reminder function supported a regular daily routine, and the usage records helped participants track their progress; 1 participant requested a more customizable reminder time.

**Table 4.** System Usability Scale (SUS) scores and open-ended feedback from the 5 app–arm participants who completed the evaluation.

Participants	SUS score^a^	User feedback
1	87.5	The video format is excellent; I do not forget the exercise content.
2	77.5	The alarm reminder function could be made more customizable.
3	90.0	I found this approach very helpful for rehabilitation.
4	92.5	It helps me perform the rehabilitation exercises consistently every day.
5	90.0	It is helpful for tracking records, and I do not forget the movements.

a Mean 87.5 (SD 5.86).

#### Clinical Outcomes (Exploratory)

Both groups improved on all clinical outcomes over the 4-week intervention. Because this was a small sample trial of 5 participants per group, clinical outcomes are presented descriptively at the individual level (Figures 4 and 5), with group means summarized in Table 2. Consistent with the exploratory framing, no inferential testing was performed, and no *P* values are reported. The magnitude and direction of the between-group differences—the contrast most relevant to whether the app outperformed the handout—are summarized in Table 2; across outcomes, the handout group showed numerically larger improvements in most measures, and no consistent advantage of the app was evident.

**Figure 4.**
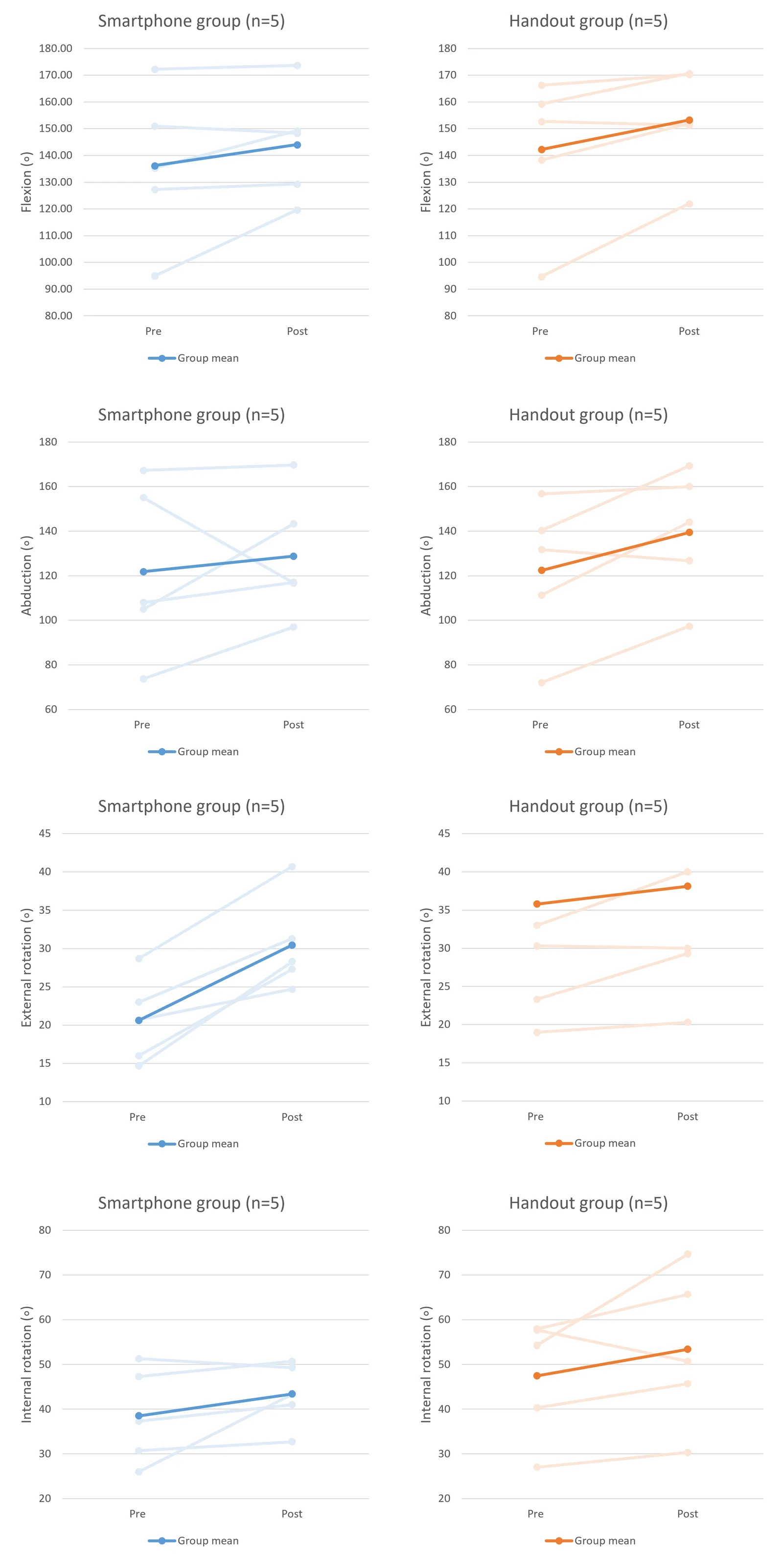
Individual and group mean shoulder range of motion trajectories (flexion, abduction, and external and internal rotation) from preintervention to postintervention over 4 weeks, by arm (app, n=5; handout, n=5); exploratory, descriptive.

**Figure 5.**
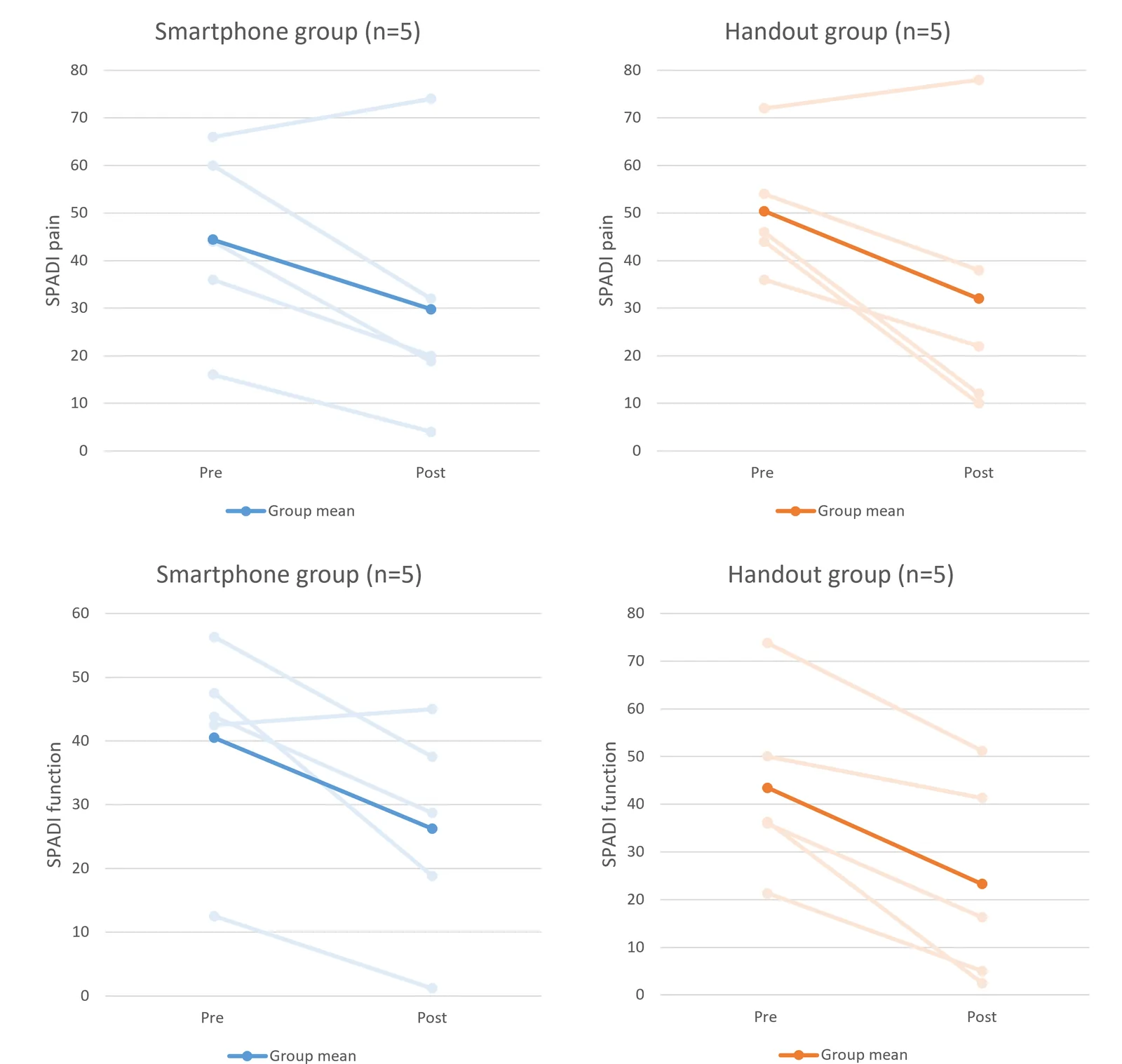
Individual and group mean Shoulder Pain and Disability Index (SPADI) pain and function trajectories from preintervention to postintervention over 4 weeks, by arm (app, n=5; handout, n=5); exploratory, descriptive.

#### Pain and Function (SPADI)

Both SPADI pain and function decreased in the 2 groups, with numerically larger improvements in the handout group (Table 2); no descriptive advantage for the app was evident.

#### Shoulder Range of Motion

Shoulder flexion, abduction, and internal rotation increased in both groups, with the handout group again showing numerically larger or comparable gains and no consistent between-group advantage (Table 2; individual trajectories in Figure 4). For shoulder external rotation, all 5 app-group participants improved (Figure 4), and the mean gain was larger in the app group (+9.9° vs +2.3°). This apparent difference must, however, be interpreted with caution: the app group started from a substantially lower baseline (20.6° vs 35.8°), and despite the larger gain, it remained lower in absolute range of motion after the intervention (30.5° vs 38.1°). The individual trajectories in Figure 4 illustrate this pattern, and no clear advantage of the app over the handout is evident once baseline is taken into account.

## Discussion

### Principal Findings

This study aimed to develop and evaluate the usability, feasibility, and exploratory clinical outcomes of a personalized smartphone app for patients with frozen shoulder. The core contribution of this work lies in system development: we successfully engineered an integrated tool that unifies sensor-based self-assessment, pain-driven exercise personalization, and objective usage logging, overcoming the limitations of prior static, one-size-fits-all interventions. The trial confirmed that this system is safe and highly usable in an unsupervised home setting. Furthermore, the app’s usage logs provided continuous, objective documentation of home exercise, a monitoring capability that printed handouts cannot offer. We also observed a descriptive trend of improvement in shoulder external rotation. Consistent with the feasibility design, this outcome is best interpreted as a hypothesis-generating signal that warrants further investigation. Ultimately, this study establishes a robust technological foundation and practical feasibility to justify a future adequately powered definitive trial.

### System Usability and Feasibility

A key strength of this trial was the confirmation that the smartphone app possesses high clinical feasibility and system usability in the population of patients with frozen shoulder. Notably, the trial achieved a 100% retention rate with no dropouts during the 4-week intervention. Furthermore, a qualitative review of the weekly follow-up communications revealed a complete absence of technical or operational troubleshooting requests; no participants reported difficulties with system manipulation, notifications, or daily usage, indicating a highly intuitive interface.

Quantitative usability assessments further supported these observations. The SUS score of the app group reached 87.5 (SD 5.86), well above the established average SUS benchmark of 68 and corresponding to an “excellent” rating on the adjective rating scale reported by Bangor et al [31]. It should be neutrally noted, however, that these SUS evaluations were provided exclusively by the app-arm participants who completed the trial. While this reflects high satisfaction among active users, it inherently excludes the perspectives of those who initially declined participation, and thus represents the usability among completers rather than universal acceptance.

This high usability rating is highly consistent with the pilot study by Stütz et al [22], which likewise developed a dedicated home-based rehabilitation app for frozen shoulder and reported an SUS score of 88. By comparison, through more comprehensive cross-validation of quantitative and qualitative feedback, the present study further confirmed the practical advantages of the system. Qualitative participant feedback indicated that the intuitive video-based exercise demonstrations effectively overcame a key limitation of traditional printed educational handouts, with which movement details are easily forgotten. Users reported that the videos helped them review movements at any time and that the built-in usage records successfully tracked their daily rehabilitation status. Regarding the exercise reminder function, participants affirmed that it helped establish a regular rehabilitation habit; additionally, specific feedback suggested that allowing users to personally customize the alarm times in future iterations could further optimize the system’s flexibility for busy schedules. Together, these findings fully confirm that this integrated system can effectively fulfill the supervisory function of home-based telerehabilitation.

### Exercise Adherence

Exercise adherence was a primary feasibility outcome. Adherence was captured with different instruments in the 2 groups: through the app’s built-in objective usage logs in the app group and through weekly interview-based self-reports in the handout group. Because these instruments are fundamentally not equivalent, adherence values cannot be compared between groups and are interpreted only as descriptive feasibility metrics. Over the 28-day program, the app group’s logs documented a mean of 18.0 (SD 5.0) exercise days (18/28, 64.3% adherence). Interpreted on its own terms, the app group’s data represent a substantial level of unsupervised home engagement and demonstrate an objective self-monitoring capability that printed handouts structurally cannot provide.

When attempting to contextualize the app group’s logged adherence rate (18/28, 64.3%) in the literature, it is evident that a directly comparable reference point is currently lacking. For instance, while Chen et al [25] reported high adherence rates (96.6% and 85.2%), their trial used daily text messages and a substantially different overall treatment approach. Furthermore, their adherence data were collected via self-report over a 2-week interval, whereas our app group relied on continuous, automated daily system logs. Given the fundamental differences in both intervention modalities and measurement timeframes, a direct numerical comparison of their findings with ours is methodologically unsuitable. Other related trials have relied on subjective numerical rating scales without calculating specific adherence percentages, precluding direct numerical comparisons [18,23]. Conversely, although Stütz et al [22] used an objective, app-based tracking mechanism similar to ours, their study was a small, single-arm evaluation (n=5). Consequently, while the app group’s logged adherence appears numerically lower than rates reported in recall-based studies, there is currently no literature comparable to our methodology that can serve as a direct, objective benchmark.

Nevertheless, this level of adherence highlights a critical clinical reality: there is a gap between theoretical effectiveness and real-world adherence. To achieve better outcomes, patients need to engage in the intervention actively, yet many factors influence adherence. For instance, patients with a 9-to-5 job may lack the time and energy to perform rehabilitation after work, and requiring daily exercise may feel pressured and overwhelming. Therefore, providing patients with a specific, practical adherence target—such as completing exercises at least 3 days per week (approximately 42% compliance)—may be more useful and realistic. Setting these targets in a progressive (graded) manner in future digital interventions may better sustain long-term patient motivation without imposing undue psychological pressure.

Furthermore, this asymmetry in measurement highlights an important methodological lesson for future research. To accurately evaluate whether a digital app improves adherence and to examine adherence as a mediator linking system use to clinical change, future definitive trials must apply a single, objective adherence measure across all arms—such as electronically time-stamped exercise logging for all participants.

### Exploratory Clinical Outcomes and System Refinement Insights

After 4 weeks of home training, both groups demonstrated descriptive improvements in pain reduction (SPADI) and shoulder range of motion (flexion, abduction, and internal rotation). For these parameters, the handout group showed numerically larger or comparable mean gains, and the descriptive data did not indicate a consistent between-group advantage for the app. As a small-sample feasibility evaluation, these descriptive trajectories align with previous short-term interventions and confirm the clinical safety of deploying the app as an add-on mHealth tool. Crucially, the absence of a clear descriptive between-group difference in overall pain and general range of motion may have been confounded by concurrent medical co-interventions. Because participants continued to receive routine care under the national health insurance system (including physical therapy modalities and pharmacological treatments), the substantial efficacy of these routine treatments may have produced a ceiling effect in the short term, obscuring the marginal benefit of the home exercise. This observation reinforces the intended positioning of the system: it serves as a reinforcing digital aid for objective home monitoring, rather than a replacement for routine medical care.

A specific descriptive pattern of practical interest emerged in shoulder external rotation, a clinically challenging movement often severely restricted and pain-provoking in frozen shoulder. All 5 app-group participants showed a consistent within-group improvement, with a numerically larger mean gain than the handout group (+9.9° vs +2.3°). While the app operationalizes a pain-stratified adjustment mechanism designed to safely guide patients through these challenging movements, this observed difference must be interpreted with strict caution. As noted previously, the app group began with a substantially lower baseline in external rotation (20.6° vs 35.8°), providing a larger window for improvement and rendering the data highly susceptible to regression to the mean. Therefore, rather than being interpreted as evidence of clinical superiority, this external-rotation pattern serves strictly as a hypothesis-generating signal to be tested in future adequately powered trials using stratified randomization to control for baseline severity.

Finally, the exploratory clinical data provide direct functional insights for future system refinement. For instance, the app group showed a more modest descriptive gain in shoulder abduction compared to the handout group (+6.3° vs +17.1°). Because the app’s current personalized algorithm adopts a highly conservative intensity to prioritize home safety, it did not emphasize reaching the extreme end range of motion during abduction exercises. This outcome highlights a clear technical direction for the next iteration of the system: refining the dynamic exercise algorithm to safely and progressively guide patients toward greater end-range loading once their tissue irritability decreases.

### Limitations

This study has several limitations. First, this study was a usability and feasibility trial, and its small overall enrollment (n=10) inherently limits the precision and generalizability of the clinical estimates. The clinical outcomes are strictly exploratory and hypothesis-generating; adequately powered randomized controlled trials are required to establish clinical efficacy. Second, the fixed-time push notifications (9 AM and 9 PM) lacked the flexibility to integrate more deeply with patients’ daily routines, potentially limiting their behavior-change potential. Third, concurrent usual care under the National Health Insurance system, including physical therapy modalities and pharmacological treatments, was not quantitatively tracked. Neither the frequency and type of physical therapy sessions nor the types and doses of medications were recorded in either group, confounding the evaluation of the smartphone intervention’s isolated clinical contribution. Future trial protocols should use a standardized weekly cointervention log for all arms (physical therapy sessions and modalities, medications and doses, and injections), ideally verified against National Health Insurance records. Fourth, the 4-week study period precludes the assessment of sustained adherence and long-term benefits, which are critical given the 1- to 2-year natural course of frozen shoulder. Fifth, prioritizing patient safety in an unsupervised setting, the current personalization algorithm stratifies intensity based solely on subjective pain. Future iterations should comprehensively integrate objective range of motion data once compensatory-movement filtering algorithms mature. Finally, this trial was not registered in a public trial registry, either prospectively or retrospectively, which reduces transparency and limits compliance with international trial reporting standards. Any future efficacy trial based on this prototype will be prospectively registered in a public trial registry.

### Conclusions

In summary, this study successfully developed and demonstrated the feasibility and usability of a telerehabilitation system for frozen shoulder that integrates several key functions, including digital education, personalized irritability-based self-assessment and stratification, objective monitoring via an SQLite database, and scheduled exercise reminders. Its core value lies in moving beyond the traditional one-size-fits-all home rehabilitation model and putting home-based precision rehabilitation into practice. The results of this study confirmed that when used as an add-on mHealth intervention to usual care, the system possesses excellent usability (SUS score of 87.5), can effectively overcome the drawback of traditional printed education—with which movements are easily forgotten—and enables objective tracking of day-to-day home exercise through its usage log. Over the 4-week home intervention, the usage logs documented home exercise on a mean of 18.0 (SD 5.0) of 28 (64.3%) days, and all app-group participants showed a within-group improvement in the most clinically challenging plane of shoulder external rotation. Because this was a small-sample trial, no inferential between-group testing was undertaken. The descriptive data, shaped by the small sample, the baseline imbalance in external rotation, and unmonitored usual care (physical therapy modalities and pharmacological treatments), should not be considered evidence of clinical superiority; nevertheless, the system demonstrated clear value in usability, feasibility, and objective home monitoring as an assistive tool for home-based telerehabilitation. Future work should expand the sample size, incorporate an intelligent personalized dynamic reminder mechanism, and combine long-term follow-up to establish its long-term evidence-based standing in the field of digital health.

## Supplementary material

10.2196/107314 Multimedia Appendix 1 Reliability and validity of range of motion assessment tool.

10.2196/107314 Multimedia Appendix 2 Training list.

10.2196/107314 Multimedia Appendix 3 Educational handout English version.

10.2196/107314 Multimedia Appendix 4 Educational handout Chinese version.

10.2196/107314 Checklist 1 CONSORT extension pilot and feasibility trials checklist.

10.2196/107314 Checklist 2 CONSORT-EHEALTH V1.6 (2011) checklist.

## Abbreviations

APTA: American Physical Therapy Association
CONSORT: Consolidated Standards of Reporting Trials
CONSORT-EHEALTH: Consolidated Standards of Reporting Trials of Electronic and Mobile Health Applications and Online Telehealth
mHealth: mobile health
SPADI: Shoulder Pain and Disability Index
SUS: System Usability Scale
