# Supplementary material for: Development, Usability, and Feasibility of a Pain-Stratified Smartphone-Based Home Intervention System for Patients With Adhesive Capsulitis: Exploratory Feasibility and Usability Trial
Source: JMIR Rehabil Assist Technol. 2026 Nov 5;13:e107314. doi: 10.2196/107314 (PMC13739270; doi:10.2196/107314)
Supplement: Supplementary file 1 [file rehab-v13-e107314-s001.pdf]

Multimedia Appendix 4. Reliability and Validity of the Sensor-Based Range-of-Motion Measurements

The smartphone application estimates shoulder range of motion from the device's built-in inertial sensors. The reliability and validity of these estimates were evaluated in a separate study by comparing the application's sensor-based measurements against universal goniometer (UG) for flexion, abduction, external rotation, and internal rotation. Agreement and reliability were quantified using the intraclass correlation coefficient (ICC), the Pearson correlation coefficient (PCC), and Lin's concordance correlation coefficient (CCC). The results are summarized in Appendix Table 1 to Table 3. Full methodological details and the complete analysis are reported separately (manuscript in preparation).

Table 1. Intra-rater reliability of smartphone measurements within-day

Measurement movements	ICC	95% CI	SEM(°)	90% MDC
Shoulder flexion	0.96	0.86-0.99	3.9	9.0
Shoulder abduction	0.97	0.88-0.99	5.0	11.6
Shoulder external rotation	0.95	0.85-0.99	3.3	7.8
Shoulder internal rotation	0.97	0.89-0.99	2.5	5.8

ICC, intraclass correlation coefficient; 95% CI, confidence interval; SEM, standard error of measurement; MDC, minimal detectable change.

Table 2. Intra-rater reliability of smartphone measurements between-day

Measurement movements	ICC	95% CI	SEM(°)
Shoulder flexion	0.84	0.50-0.95	8.7
Shoulder abduction	0.83	0.50-0.95	10.6
Shoulder external rotation	0.95	0.84-0.99	3.4
Shoulder internal rotation	0.90	0.65-0.97	3.9

ICC, intraclass correlation coefficient; 95% CI, confidence interval; SEM, standard error of measurement.

Table 3. Validity of smartphone measurements

Measurement movements	PCC	*p value*	CCC
Shoulder flexion	0.99	<.01	0.98
Shoulder abduction	0.96	<.01	0.95
Shoulder external rotation	0.91	<.01	0.84
Shoulder internal rotation	0.95	<.01	0.92

PCC, Pearson's correlation coefficient; CCC, concordance correlation coefficient.
