# Supplementary material for: Development, Usability, and Feasibility of a Pain-Stratified Smartphone-Based Home Intervention System for Patients With Adhesive Capsulitis: Exploratory Feasibility and Usability Trial
Source: JMIR Rehabil Assist Technol. 2026 Nov 5;13:e107314. doi: 10.2196/107314 (PMC13739270; doi:10.2196/107314)
Supplement: Supplementary file 3 [file rehab-v13-e107314-s003.pdf]

Appendix 1: Intervention Exercise List

This study referenced prior literature on frozen shoulder exercises. The intervention required participants in both groups to perform the same home-based exercises. The exercise intensity was categorized into three levels, the details of which are described below.

A. Low-Intensity Exercise

Exercises should be performed within a pain-free range, at least once a day, for at least 15 minutes per session, as shown in Table 1.

Table 1: Low-Intensity Exercise Items and Contents

Exercise Item	Video Content
1. Correct Posture Reminders	Demonstrates precautions for maintaining correct sitting, supine sleeping, and side-lying sleeping postures in daily life.
2. Pendulum Exercise	Support the body weight by placing the elbow of the unaffected side on a stable object (e.g., a table or chair). Keep the back straight without slouching, and let the affected arm hang naturally. Use body sway to drive the forward and backward swinging motion of the arm for approximately 5 minutes.
3. Neck Stretching Exercise	Hold the chair with the hand of the affected side. Tilt the ear of the unaffected side toward the shoulder of the unaffected side until a stretch is felt in the affected shoulder and neck. Then, turn the head toward the unaffected side until a slight tightness is felt. Hold for 15–30 seconds. Repeat at least 5 times.
4. Arm Elevation Exercise	Hold an umbrella or a long stick with both hands. Raise the affected arm within a pain-free range. Inhale while slowly raising the arm with assistance from the unaffected hand, and exhale while slowly lowering it. Repeat at least 10 times.
5. Arm Abduction Exercise	Hold an umbrella or a long stick with both hands, with the palm of the affected hand facing forward and the thumb pointing up (thumbs-up position). Raise the affected arm within a pain-free range. Inhale while slowly raising the arm with assistance from the

(Scaption)	unaffected hand, and exhale while slowly lowering it. Avoid shrugging the shoulders. Repeat at least 10 times.
6. Arm External Rotation Exercise	Hold an umbrella or a long stick with both hands. Flex the affected elbow to 90 degrees and place a rolled towel between the upper arm and the torso. Within a pain-free range, perform an outward "door-opening" motion with the affected arm, using the unaffected hand to assist by pushing. Repeat at least 10 times.
7. Hand-Behind-Back Exercise	Hold an umbrella or a long stick with both hands, placing the affected hand below and the unaffected hand above. Within a pain-free range, move the affected hand upward behind the back, using the unaffected hand above to assist by pulling upward. Repeat at least 10 times.

B. Medium-Intensity Exercise

Exercises should be performed at least once a day, for at least 20 minutes per session, as shown in Table 2.

Table 2: Medium-Intensity Exercise Items and Contents

Exercise Item	Video Content
1. Correct Posture Reminders	Demonstrates precautions for maintaining correct sitting, supine sleeping, and side-lying sleeping postures in daily life.
2. Pendulum Exercise	Support the body weight by placing the elbow of the unaffected side on a stable object (e.g., a table or chair). Keep the back straight without slouching, and let the affected arm hang naturally. Use body sway to drive the forward and backward swinging motion of the arm for approximately 5 minutes.
3. Neck Stretching Exercise	Hold the chair with the hand of the affected side. Tilt the ear of the unaffected side toward the shoulder of the unaffected side until a stretch is felt in the affected shoulder and neck. Then, turn the head toward the unaffected side until a slight tightness is felt. Hold for 15–30 seconds. Repeat at least 5 times.
4. Scapular Depression Exercise	Sit upright without slouching, pressing both fists firmly downward against the bed surface. During this movement, depress and retract (squeeze inward) the scapulae

	simultaneously. Hold the retraction for 3–5 seconds before releasing. Repeat at least 10 times.
5. Serratus Anterior Strengthening Exercise	Lie in a supine position and raise the affected arm to approximately 90 degrees. Reach and extend the hand toward the ceiling so that the scapula lifts off the bed surface. Hold this position for 3–5 seconds, then relax and return. Repeat at least 10 times.
6. Middle and Lower Trapezius Strengthening Exercise	Lie on the side with knees bent to stabilize the body. Flex the affected elbow to 90 degrees, keeping the upper arm close to the side of the body. Perform a "door-opening" motion while simultaneously retracting the scapula inward. Hold the contraction for 3–5 seconds before releasing. Repeat at least 10 times.
7. Arm Elevation Stretching Exercise	Hold an umbrella or a long stick with both hands and raise the affected arm until a tight sensation is felt in the shoulder. Hold for 15–20 seconds, then repeat. Repeat at least 5 times.
8. Arm Abduction Stretching Exercise	Hold an umbrella or a long stick with both hands, with the palm of the affected hand facing forward and the thumb pointing up (thumbs-up position). Raise the affected arm until a tight sensation is felt in the shoulder. Hold for 15–20 seconds, then repeat. Avoid shrugging the shoulders. Repeat at least 5 times.
9. Arm External Rotation Stretching Exercise	Hold an umbrella or a long stick with both hands. Flex the affected elbow to 90 degrees and hold a towel between the upper arm and the torso. Perform a "door-opening" motion with the affected arm until a tight sensation is felt in the shoulder. Hold for 15–20 seconds. Repeat at least 5 times.
10. Hand-Behind-Back Stretching Exercise	Hold an umbrella or a long stick with both hands, placing the affected hand below and the unaffected hand above. Move the affected hand upward behind the back until a tight sensation is felt in the shoulder. Hold for 15–20 seconds, using the unaffected hand above to assist by pulling upward. Repeat at least 5 times.

C. High-Intensity Exercise

Exercises should be performed at least once a day, for at least 20 minutes per session, as shown in Table 3.

Table 3: High-Intensity Exercise Items and Contents

Exercise Item	Video Content
1. Correct Posture Reminders	Demonstrates precautions for maintaining correct sitting, supine sleeping, and side-lying sleeping postures in daily life.
2. Pendulum Exercise	Support the body weight by placing the elbow of the unaffected side on a stable object (e.g., a table or chair). Keep the back straight without slouching, and let the affected arm hang naturally. Use body sway to drive the forward and backward swinging motion of the arm for approximately 5 minutes.
3. Neck Stretching Exercise	Hold the chair with the hand of the affected side. Tilt the ear of the unaffected side toward the shoulder of the unaffected side until a stretch is felt in the affected shoulder and neck. Then, turn the head toward the unaffected side until a slight tightness is felt. Hold for 15–30 seconds. Repeat at least 5 times.
4. Scapular Depression Exercise	Sit upright without slouching, pressing both fists firmly downward against the bed surface. During this movement, depress and retract (squeeze inward) the scapulae simultaneously. Hold the retraction for 3–5 seconds before releasing. Repeat at least 10 times.
5. Serratus Anterior Strengthening Exercise	Lie in a supine position and raise the affected arm to approximately 90 degrees. Reach and extend the hand toward the ceiling so that the scapula lifts off the bed surface. Hold this position for 3–5 seconds, then relax and return. Repeat at least 10 times.
6. Middle and Lower Trapezius Strengthening Exercise	Lie on the side with knees bent to stabilize the body. Flex the affected elbow to 90 degrees, keeping the upper arm close to the side of the body. Perform a "door-opening" motion while simultaneously retracting the scapula inward. Hold the contraction for 3–5 seconds before releasing. Repeat at least 10 times.

7. Arm Elevation Stretching Exercise	Hold an umbrella or a long stick with both hands and raise the affected arm until a tight sensation is felt in the shoulder. Hold for 15–30 seconds, then repeat. Repeat at least 5 times.
8. Arm Abduction Stretching Exercise	Hold an umbrella or a long stick with both hands, with the palm of the affected hand facing forward and the thumb pointing up (thumbs-up position). Raise the affected arm until a tight sensation is felt in the shoulder. Hold for 15–30 seconds, then repeat. Avoid shrugging the shoulders. Repeat at least 5 times.
9. Arm External Rotation Stretching Exercise	Hold an umbrella or a long stick with both hands. Flex the affected elbow to 90 degrees and hold a towel between the upper arm and the torso. Perform a "door-opening" motion with the affected arm until a tight sensation is felt in the shoulder. Hold for 15–30 seconds. Repeat at least 5 times.
10. Hand-Behind-Back Stretching Exercise	Hold an umbrella or a long stick with both hands, placing the affected hand below and the unaffected hand above. Move the affected hand upward behind the back until a tight sensation is felt in the shoulder. Hold for 15–30 seconds, using the unaffected hand above to assist by pulling upward. Repeat at least 5 times.
