# Supplementary material for: Development, Usability, and Feasibility of a Pain-Stratified Smartphone-Based Home Intervention System for Patients With Adhesive Capsulitis: Exploratory Feasibility and Usability Trial
Source: JMIR Rehabil Assist Technol. 2026 Nov 5;13:e107314. doi: 10.2196/107314 (PMC13739270; doi:10.2196/107314)

冰凍肩衛教單

一、 什麼是冰凍肩？

冰凍肩又稱五十肩，學名為沾黏性肩關節囊炎，會導致病患的肩膀出現疼痛以及活動度的缺損，進而限制肩膀主動關節活動度及被度關節之活動度。冰凍肩的患者佔一般總人口的百分之二到百分之五，好發於四十到六十五歲的成年人，其中以女性、糖尿病、甲狀腺相關疾病患者的罹患風險機率更高。冰凍肩患者的臨床症狀會隨著病程而有所不同，前期患者在肩膀有明顯的發炎疼痛，尤其是在晚上；後期疼痛會慢慢減緩，主要的症狀為肩膀僵硬、肩關節活動度出現受限，尤其以肩關節外轉動作受限程度最大。而上述生理上的限制會導致病患無法正常工作、生活品質、睡眠及龐大的治療費用。

二、 冰凍肩的原因

引起冰凍肩的原因可分為原發性與次發性。原發性冰凍肩大部分為原因不明造成肩關節慢性發炎所致。次發性冰凍肩則是由於其他病理狀況，導致肩關節發炎。常見於中風病人的患側肩膀、上肢手術、上肢脫臼、旋轉肌受損或骨折的病人也常因疼痛、不敢活動肩膀，而導致冰凍肩。

三、 冰凍肩的分期

冰凍肩分為三個時期：第一是「疼痛期（急性期）」，症狀為、肩部急性疼痛，患者常因疼痛而不敢進行主動式活動，夜間睡覺常會痛醒。第二是「結凍期（沾黏起始期）」，受傷的組織以及關節囊的纖維由於不動關始沾黏。此時被動與主動的活動都會受到限制，疼痛會緩解但仍會發生在動作中。第三是「解凍期」，這個時期疼痛大部分會發生在動作最大範圍的末端，關節沾黏的情況會逐漸緩解。

四、 冰凍肩的治療

目前對冰凍肩有幫助的治療包含類固醇注射、儀器治療（短波、電刺激、超音波）搭配治療性的運動、徒手治療及生活中的衛教。其中類固醇注射較用於初期緩解急性疼痛；後期的治療較著重在治療性運動，治療性運動包含牽拉關節運動、主動活動度運動，能拉開沾黏的組織增加關節活動度，但由於運動時可能會造成原本沾黏的組織產生發炎，因此運動時可搭配止痛藥，並與治療師討論適合的運動頻率及強度。

五、 運動內容

低強度運動：在不痛範圍下運動，每天至少一次，每次至少

15 分鐘。

1. 正確姿勢提醒：

A. 坐姿或站姿：微縮下巴及腹部，不駝背。

B. 正躺休息時：可以在患側肩膀下墊一層被子或薄枕頭，高度不用太高，有支撐感覺即可。

C. 側躺休息時：勿躺在患側，可以在好側放枕頭或棉被給予患側支撐。

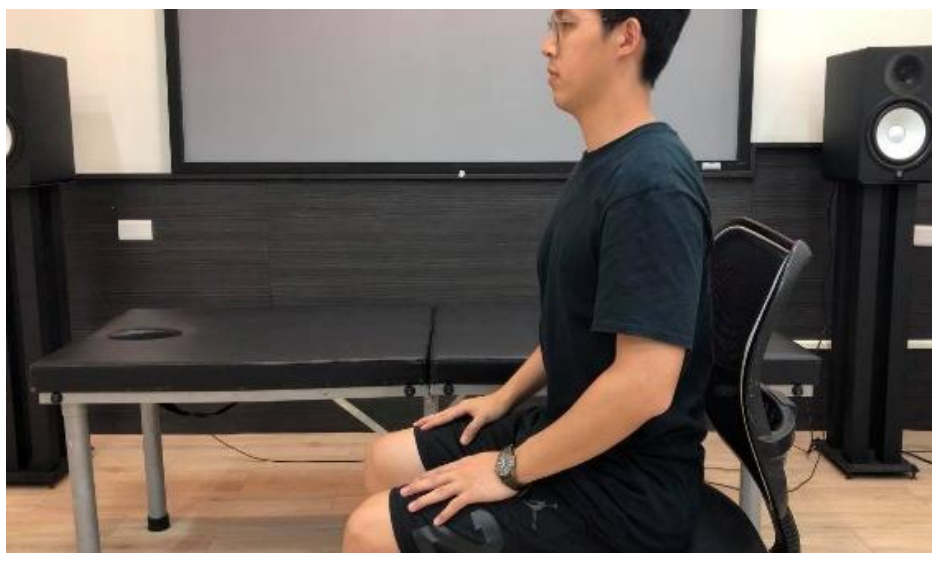
（正確坐姿）

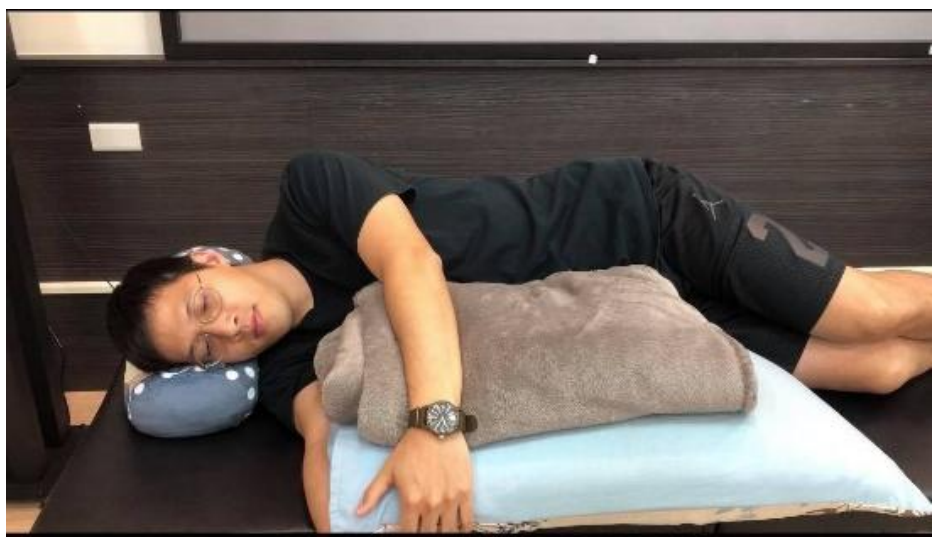
（正確側躺）

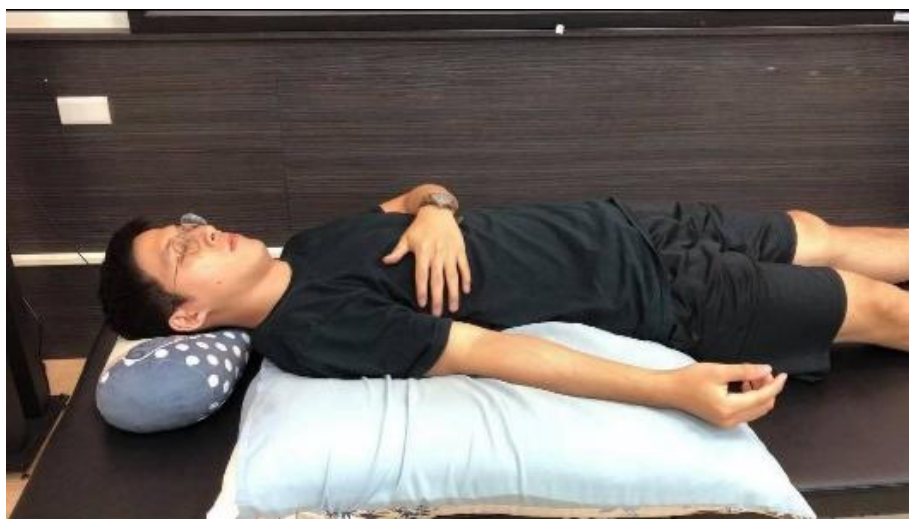
（正確正躺）

2. 鐘擺運運：

A. 用健側手肘支撐在穩定物上例如桌子或椅子。

B. 身體不彎腰，腰背一直線，患側手自然下垂

C. 用身體擺動來帶動手臂前後擺動，時間約 5 分鐘。

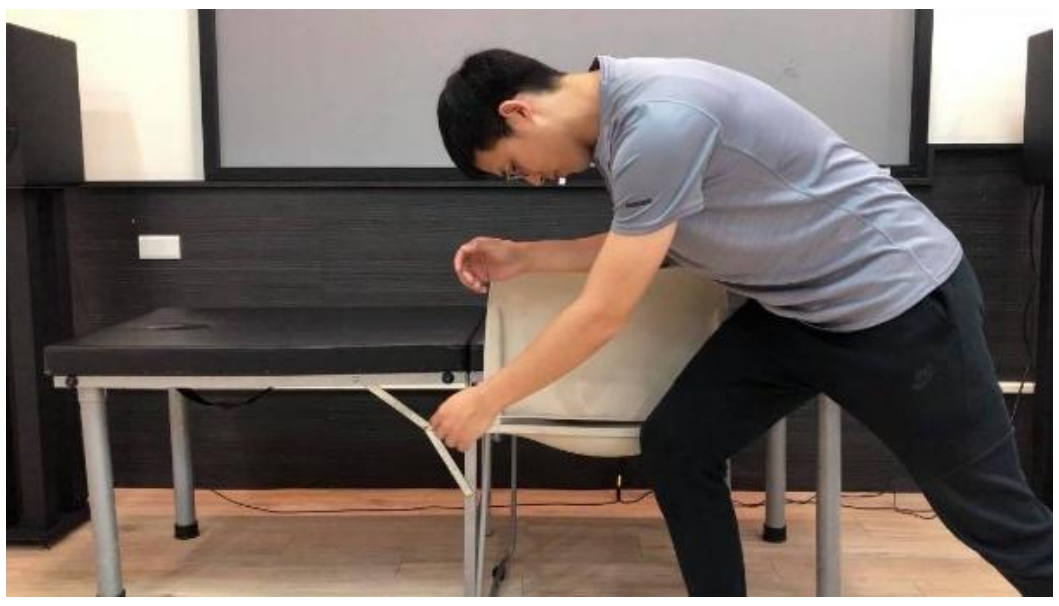

3. 頸部伸展運動：

A. 患側手抓住椅子，好邊耳朵往好邊肩膀靠。

B. 靠到患側肩頸有緊的感覺後，頭再向好邊轉。

C. 轉到微緊的感覺後，維持 15～30 秒，至少做 5 次。

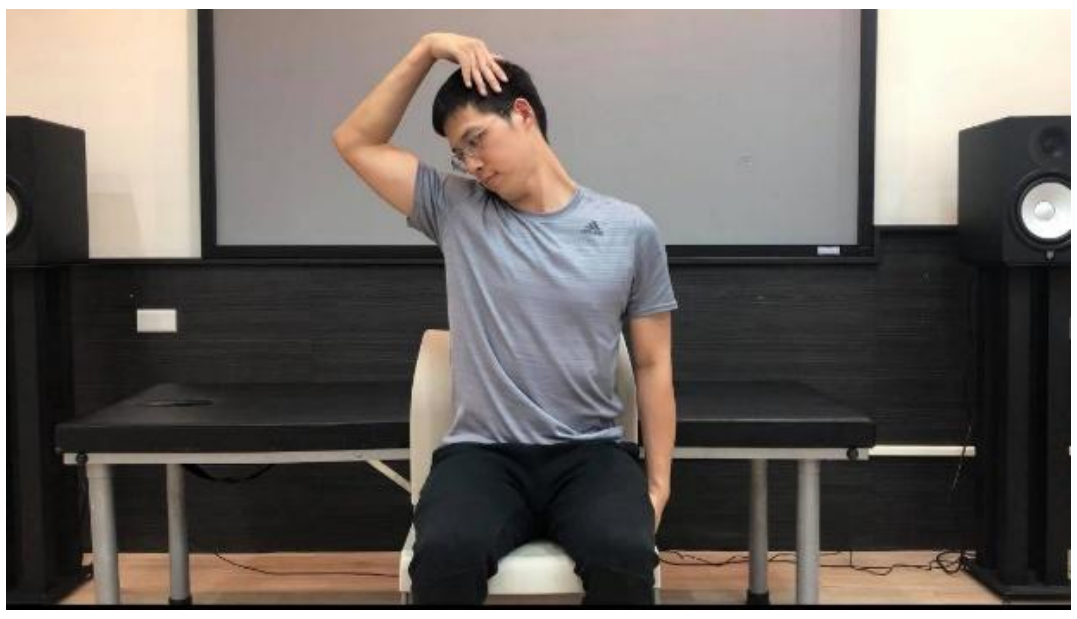

4. 手臂抬高運動：

A. 雙手握住雨傘或長棍。

B. 在不痛範圍內，患側手臂抬高，吸氣時慢慢抬高，好手輔助向上，吐氣時慢慢放下，至少做 10 次。

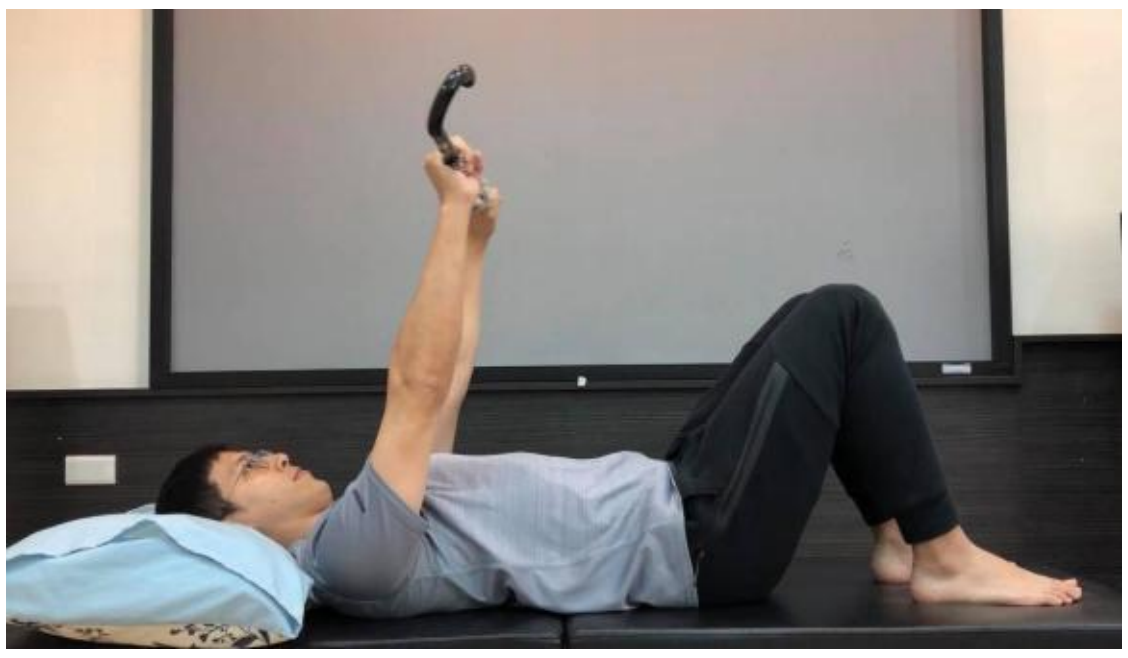

5. 手臂側邊抬高運動：

   A. 雙手握住雨傘或長棍。

   B. 患側手掌朝前比出讚。

   C. 在不痛範圍內，患側手臂抬高，吸氣時慢慢抬高，好手輔助向上，吐氣時慢慢放下，注意不聳肩，至少做 10 次。

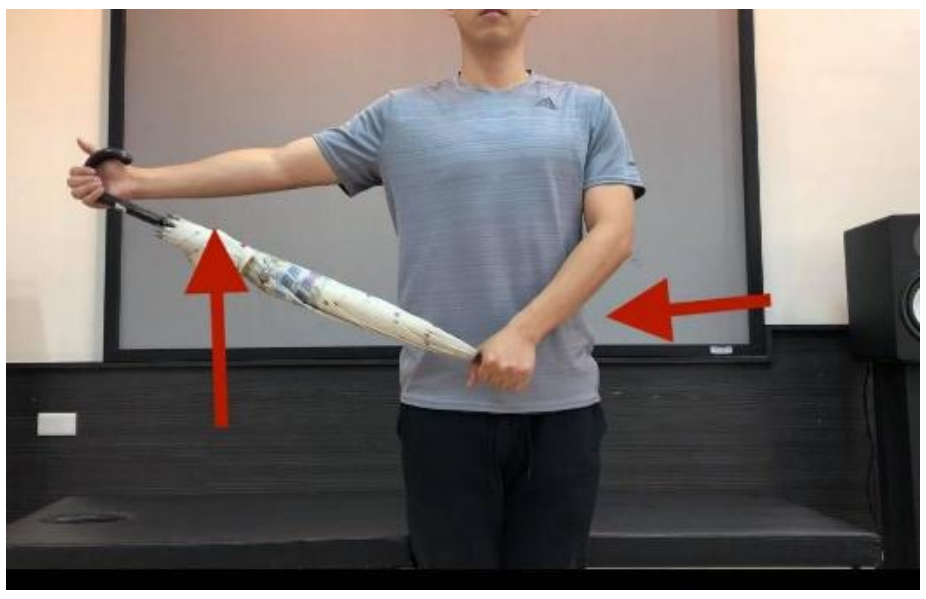

6. 手臂外轉運動：

   A. 雙手握住雨傘或長棍。

   B. 患側手臂彎曲 90 度，上臂夾著毛巾。

   C. 在不痛範圍內，患側手臂做出類似開門動作，好手可以

幫忙推，至少做 10 次。

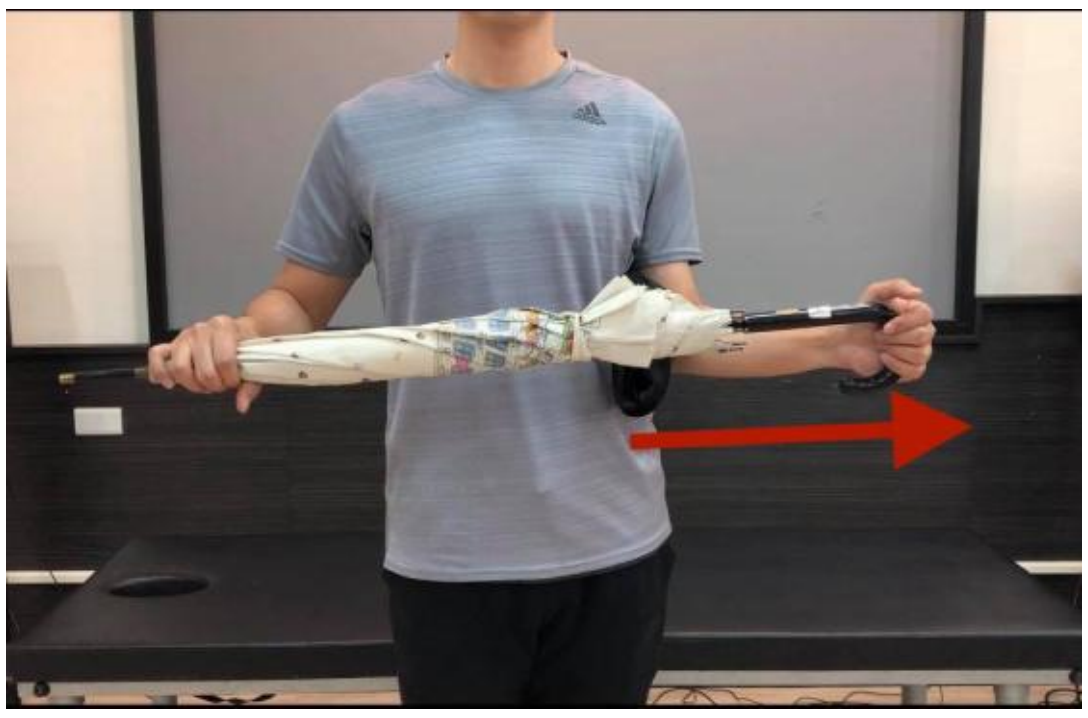

7. 摸背運動：

A. 雙手握住雨傘或長棍。

B. 患側手在下，好側手在上。

C. 在不痛範圍內，患側手臂向上摸背，好手在上幫忙拉，至少做 10 次。

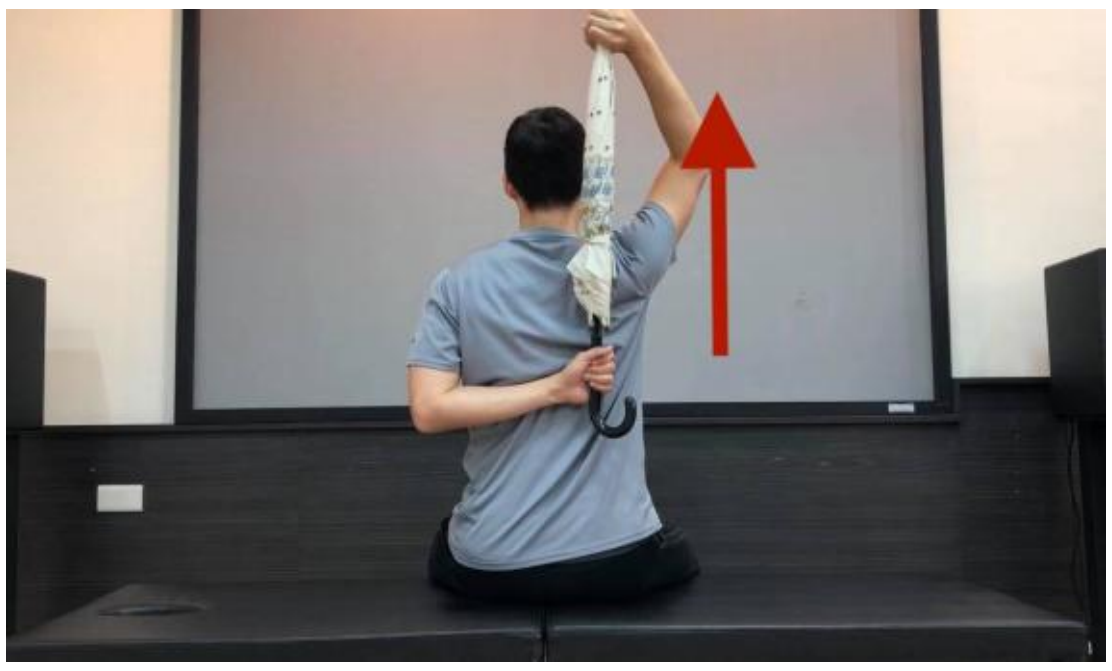

中強度運動：每天至少一次，每次至少 20 分鐘。

1. 正確姿勢體醒：

   A. 坐姿或站姿：微縮下巴及腹部，不駝背。

   B. 正躺休息時：可以在患側肩膀下墊一層被子或薄枕頭，高度不用太高，有支撐感覺即可。

   C. 側躺休息時：勿躺在患側，可以在好側放枕頭或棉被給予患側支撐。

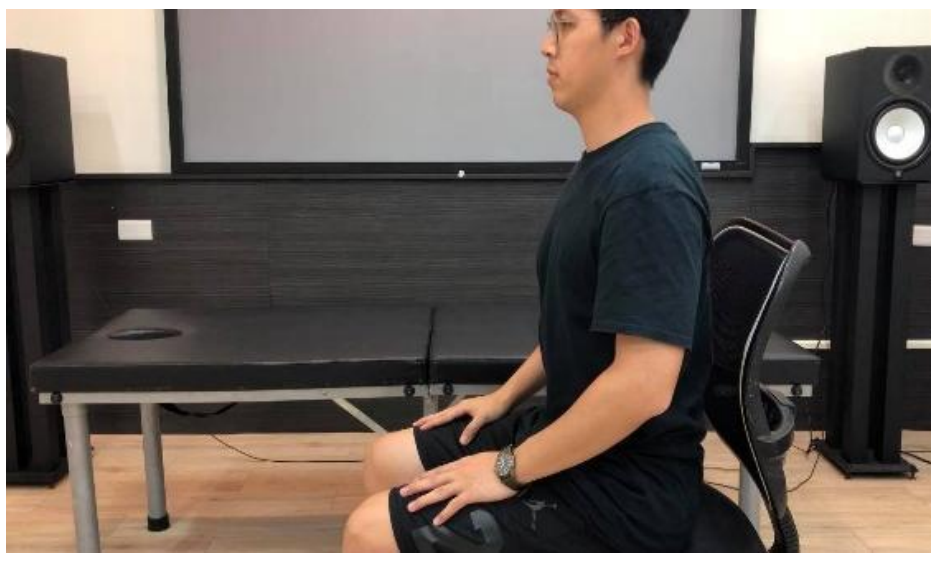

（正確坐姿）

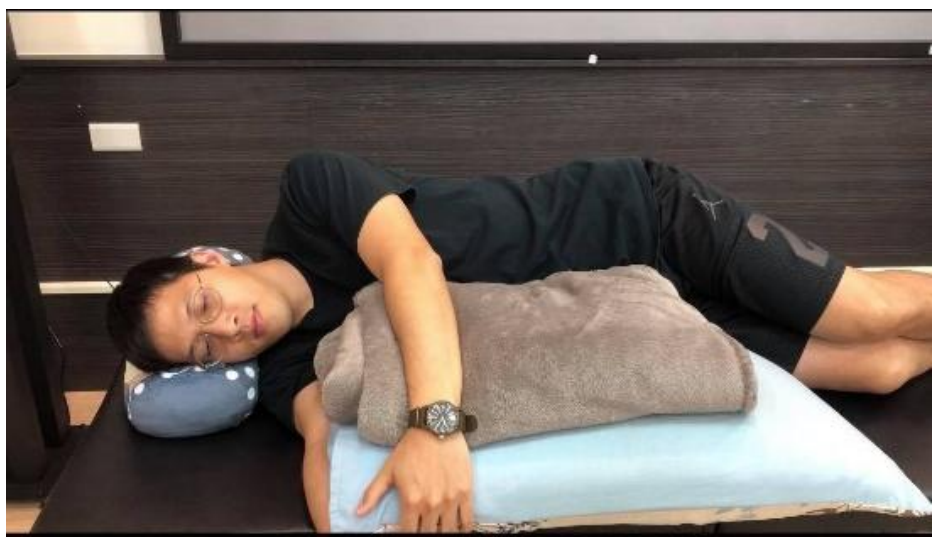

（正確側躺）

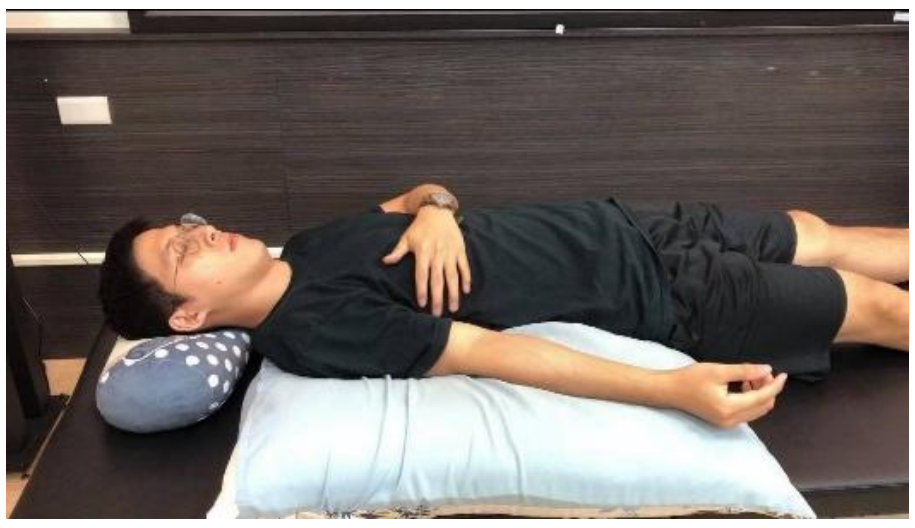

（正確正躺）

2. 鐘擺運運：

   A. 用健側手肘支撐在穩定物上例如桌子或椅子。

   B. 身體不彎腰，腰背一直線，患側手自然下垂

C. 用身體擺動來帶動手臂前後擺動，時間約 5 分鐘。

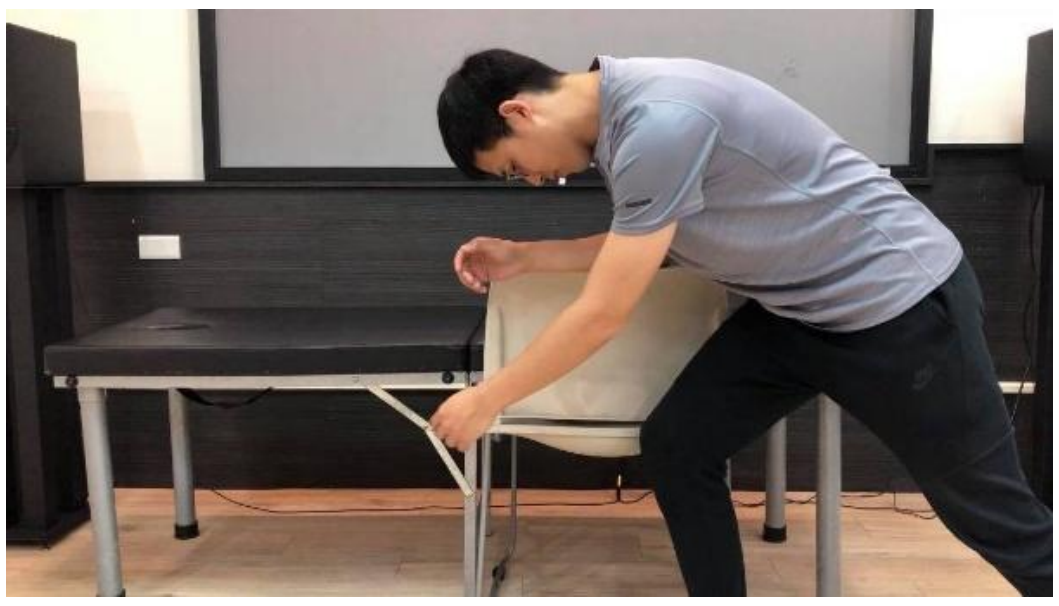

3. 頸部伸展運動：

A. 患側手抓住椅子，好邊耳朵往好邊肩膀靠。

B. 靠到患側肩頸有緊的感覺後，頭再向好邊轉。

C. 轉到微緊的感覺後，維持 15～30 秒，至少做 5 次。

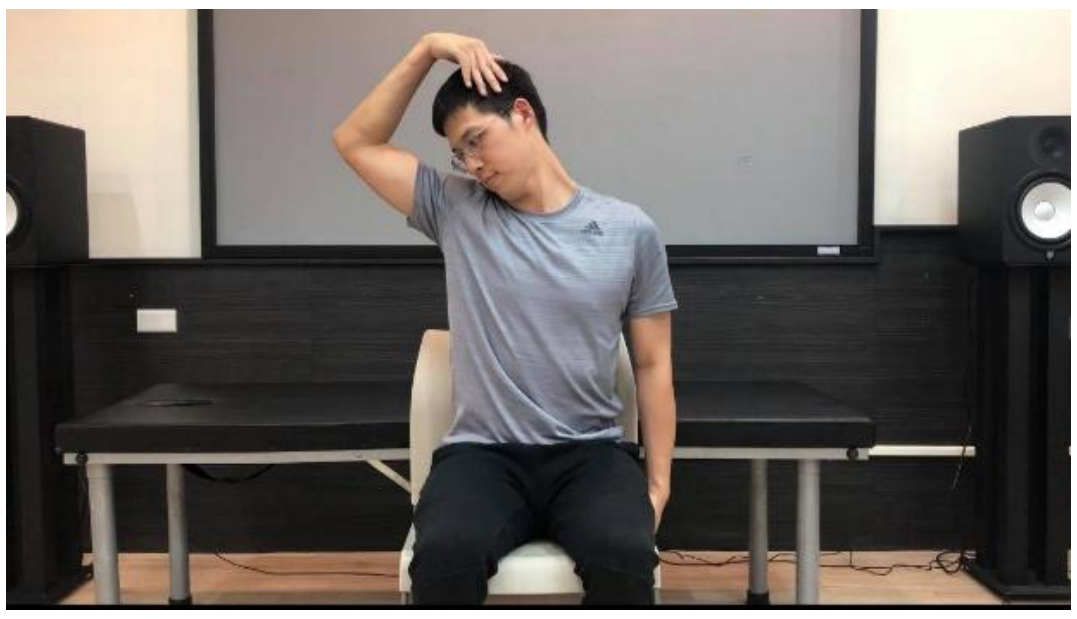

4. 肩胛骨下壓運動：

A. 坐姿不駝背，雙手拳頭頂住床面。

B. 動作時，雙手拳頭用力向下頂，同時肩胛骨向下壓向內夾。

C. 內夾 3～5 秒後再重複，至少做 10 次。

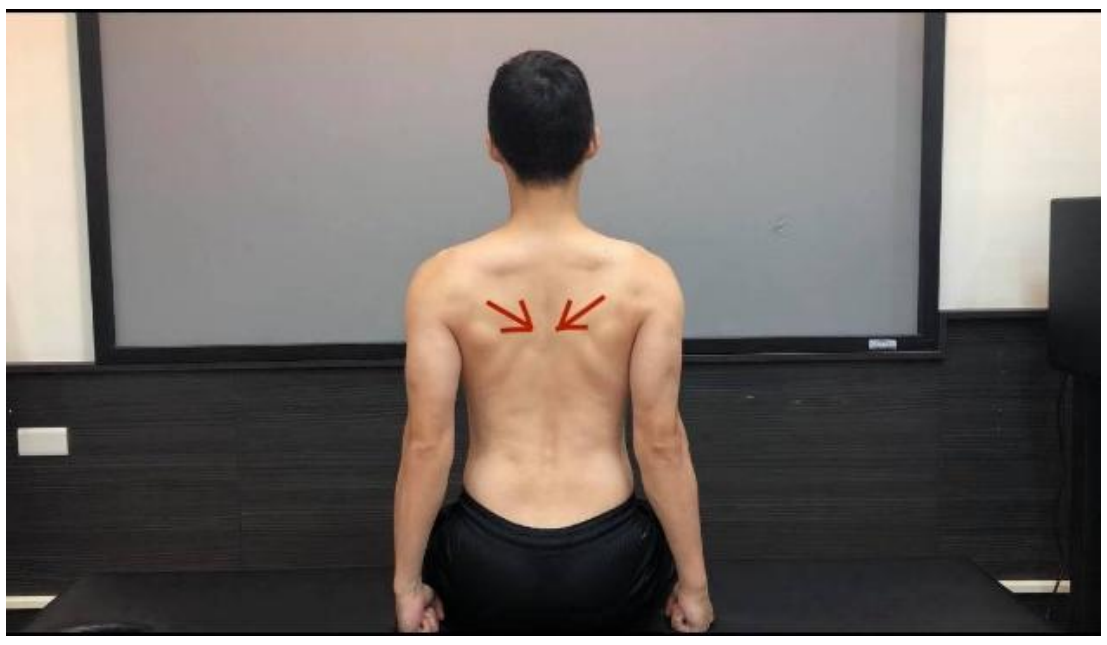

5. 前鋸肌強化運動：

   A. 躺姿，患側手臂向上舉到約 90 度。

   B. 手向天花板的方向碰及延伸，此時肩胛骨會離開床面。

   C. 用力 3～5 秒後放鬆，再重複，至少做 10 次。

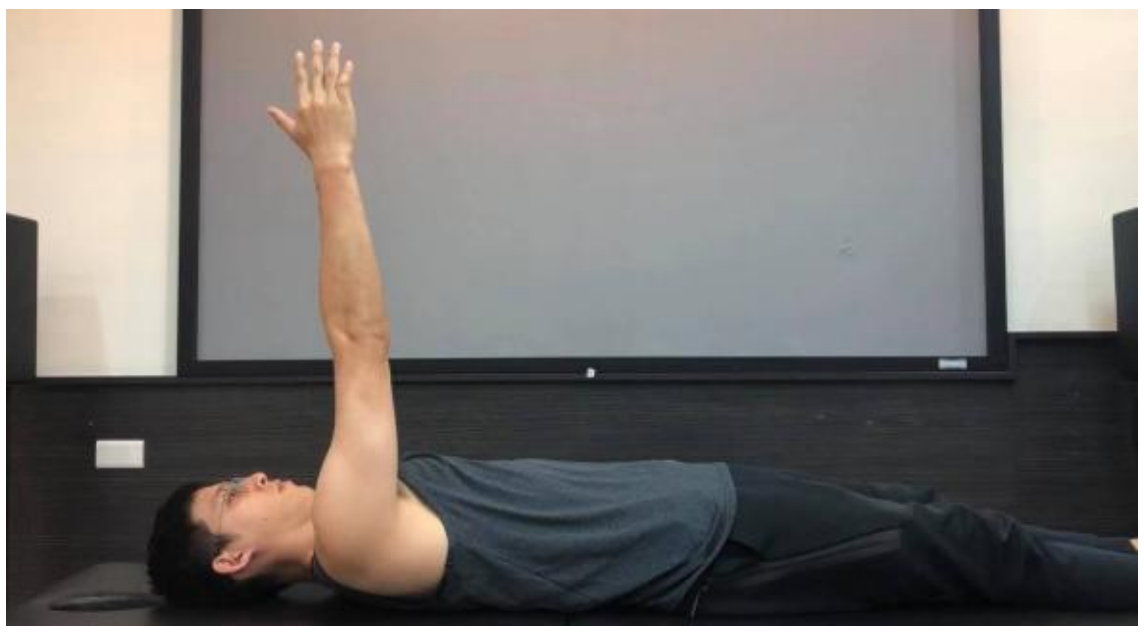

6. 中下斜方肌強化運動：

   A. 側躺，膝蓋彎曲穩定身體。

   B. 壞邊手臂彎曲九十度，上臂貼在身體旁。

   C. 動作時做出開門動作，同時肩胛骨向內夾，用力 3～5 秒再重複，至少做 10 次。

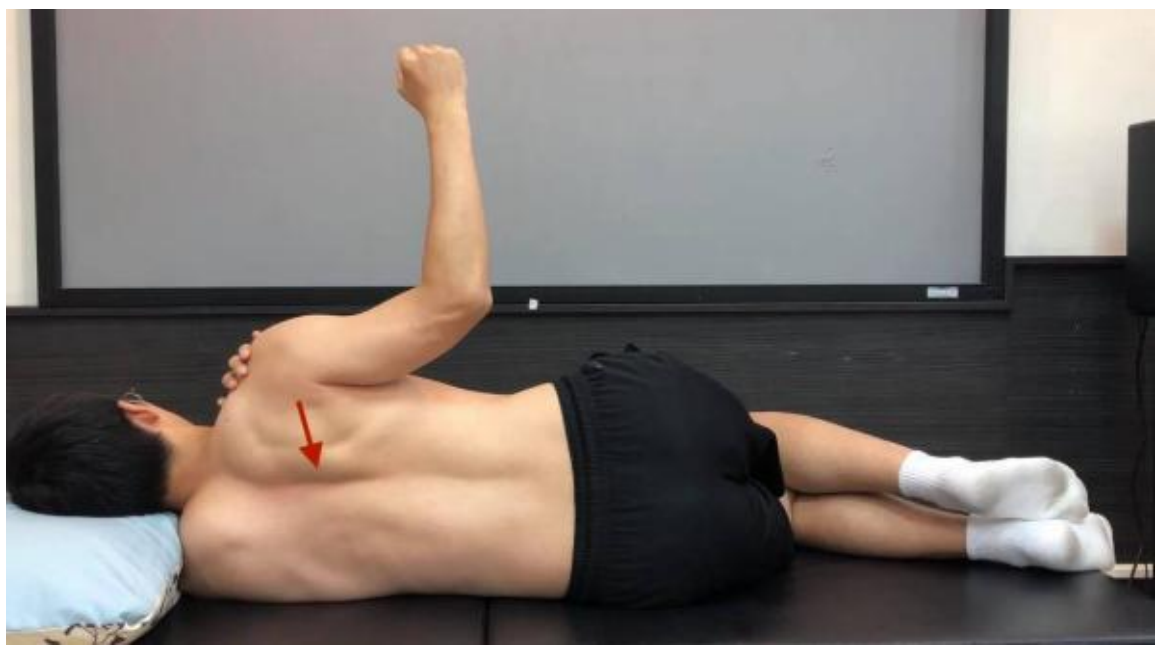

7. 手臂抬高伸展運動：

   A. 雙手握住雨傘或長棍。

   B. 患側手臂抬高，抬高到肩膀有緊的感覺，維持 15～20 秒，再重複，至少做 5 次。

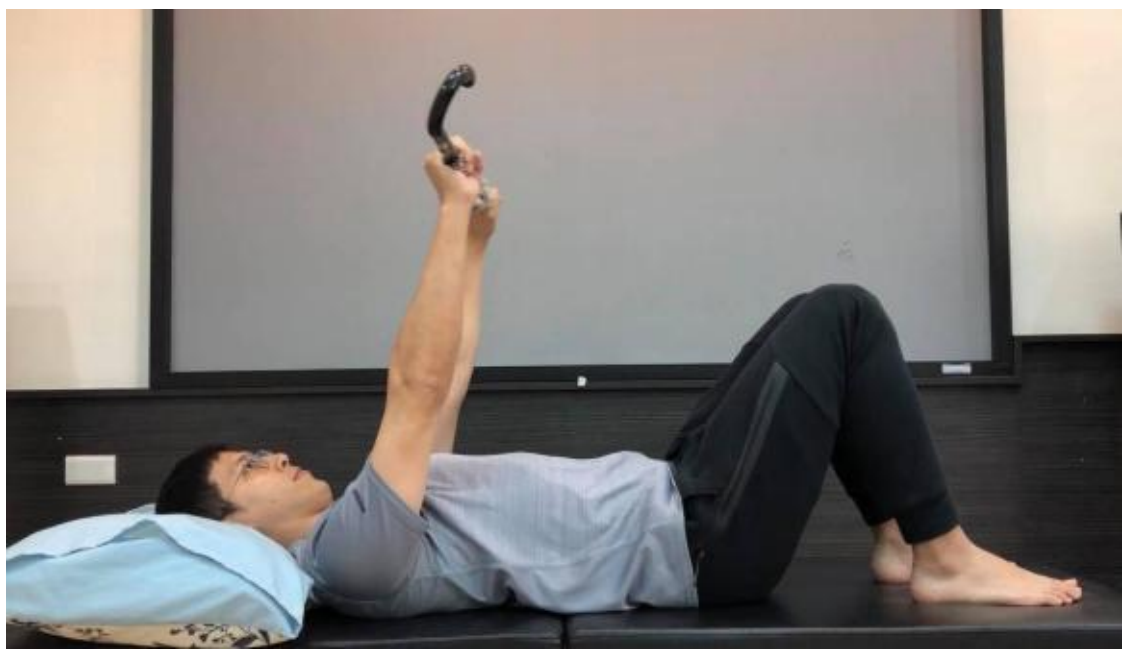

8. 手臂側邊抬高伸展：

   A. 雙手握住雨傘或長棍，患側手掌朝前比出讚。

   B. 患側手臂抬高，抬高到肩膀有緊的感覺，維持 15～20 秒，再重複，注意不聳肩，至少做 5 次。

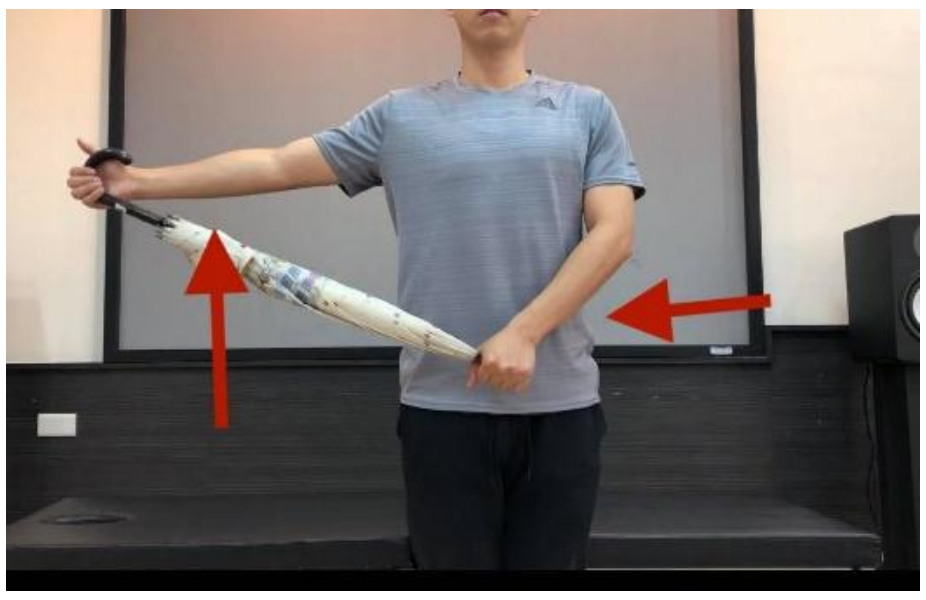

9. 手臂外轉伸展運動：

   A. 雙手握住雨傘或長棍。

   B. 患側手臂彎曲 90 度，上臂夾著毛巾。

   C. 患側手臂做出類似開門動作，開到肩膀有緊的感覺，維持 15～20 秒，至少做 5 次。

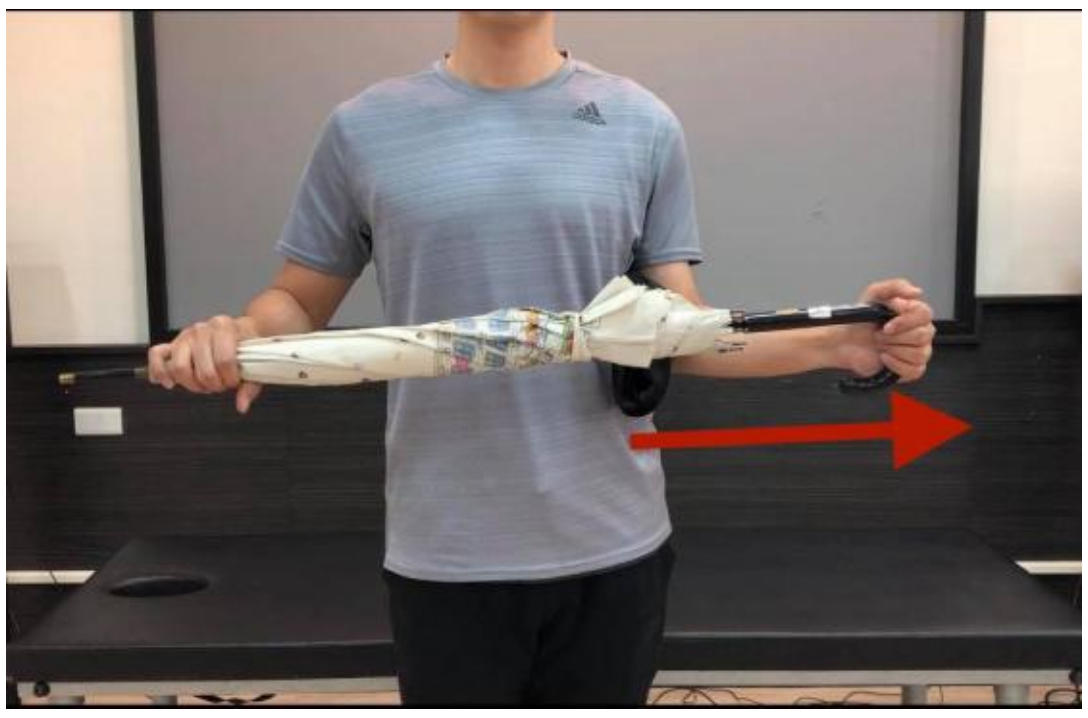

10. 摸背伸展運動：

   A. 雙手握住雨傘或長棍。

   B. 患側手在下，好側手在上。

   C. 患側手臂向上摸背，摸到肩膀有緊的感覺，維持 15～20 秒，好手可以幫忙向上拉，至少做 5 次。

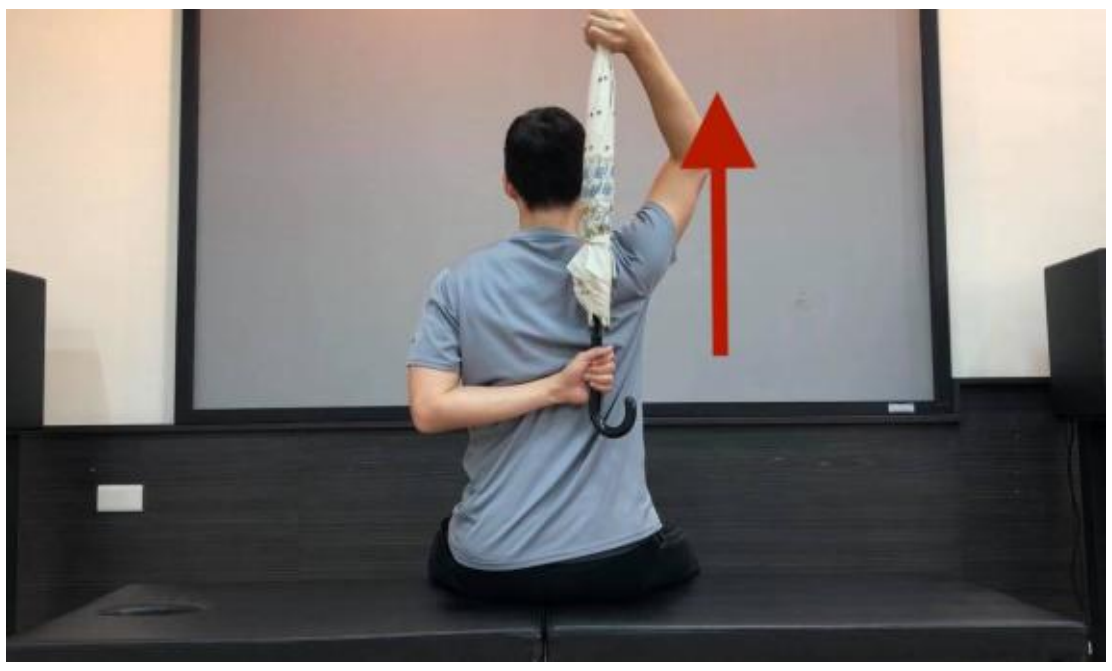

高強度運動：每天至少一次，每次至少 20 分鐘。

1. 正確姿勢體醒：

   A. 坐姿或站姿：微縮下巴及腹部，不駝背。

   B. 正躺休息時：可以在患側肩膀下墊一層被子或薄枕頭，高度不用太高，有支撐感覺即可。

   C. 側躺休息時：勿躺在患側，可以在好側放枕頭或棉被給予患側支撐。

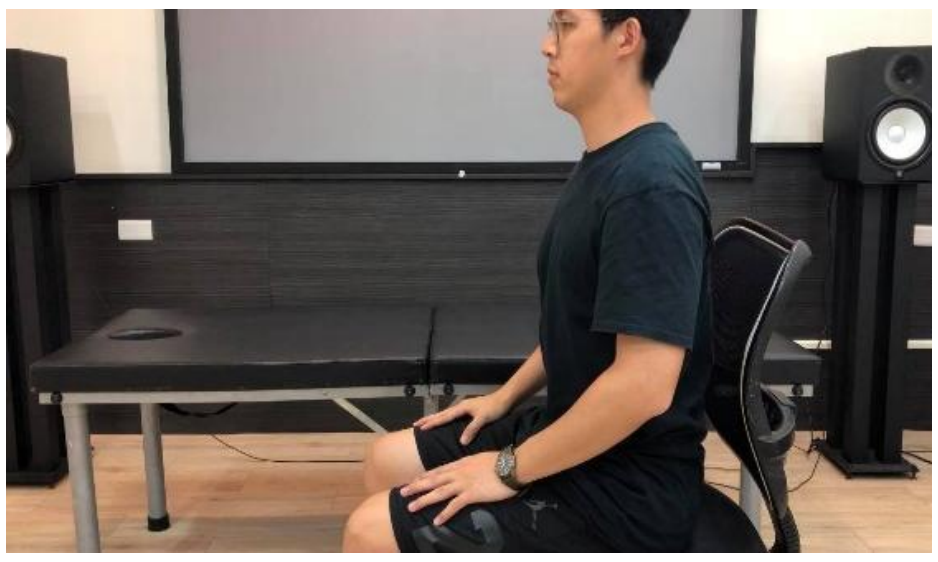

（正確坐姿）

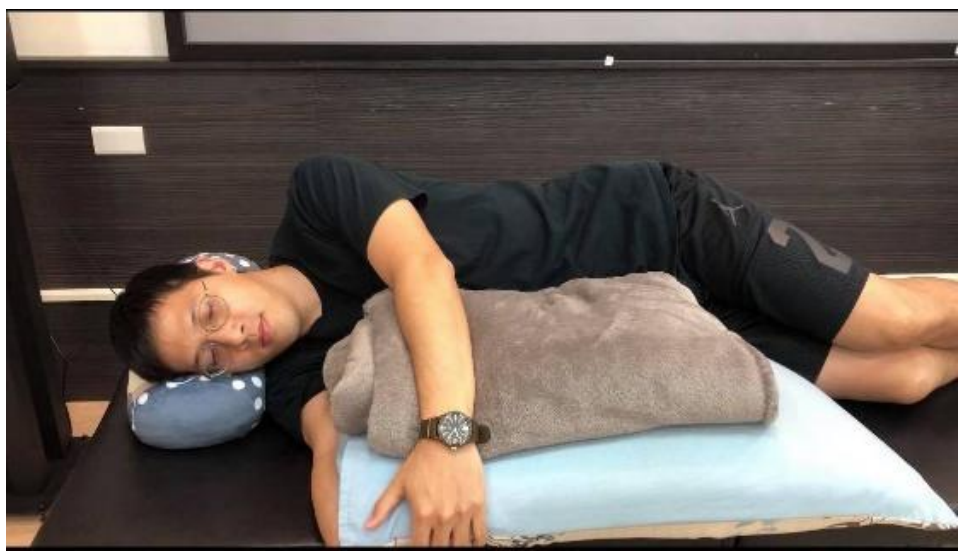

（正確側躺）

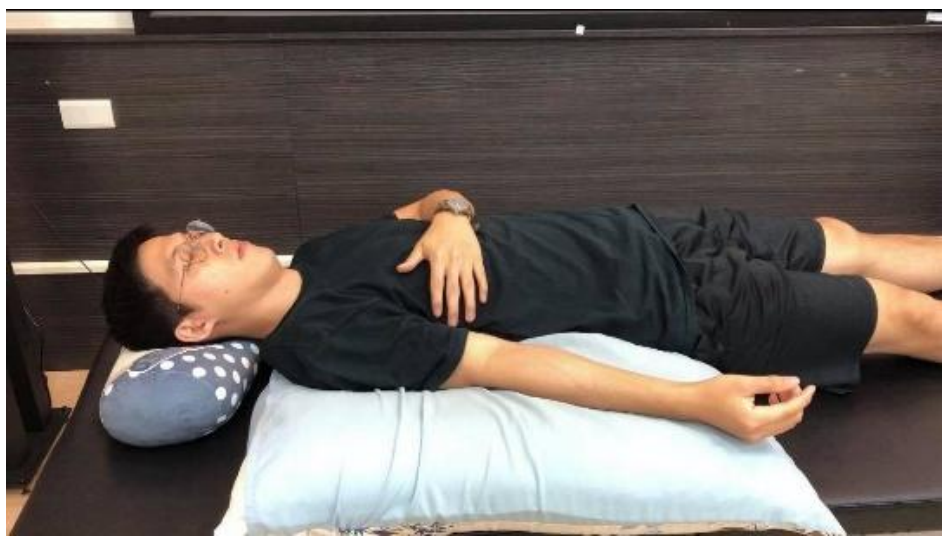

（正確正躺）

2. 鐘擺運運：

   A. 用健側手肘支撐在穩定物上例如桌子或椅子。

   B. 身體不彎腰，腰背一直線，患側手自然下垂

C. 用身體擺動來帶動手臂前後擺動，時間約 5 分鐘。

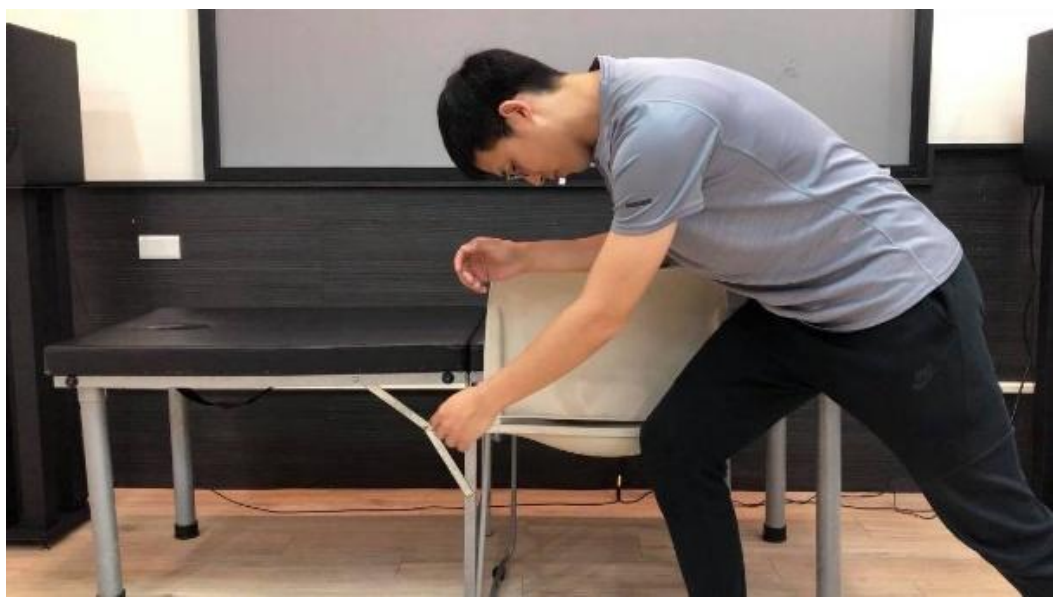

3. 頸部伸展運動：

A. 患側手抓住椅子，好邊耳朵往好邊肩膀靠。

B. 靠到患側肩頸有緊的感覺後，頭再向好邊轉。

C. 轉到微緊的感覺後，維持 15～30 秒，至少做 5 次。

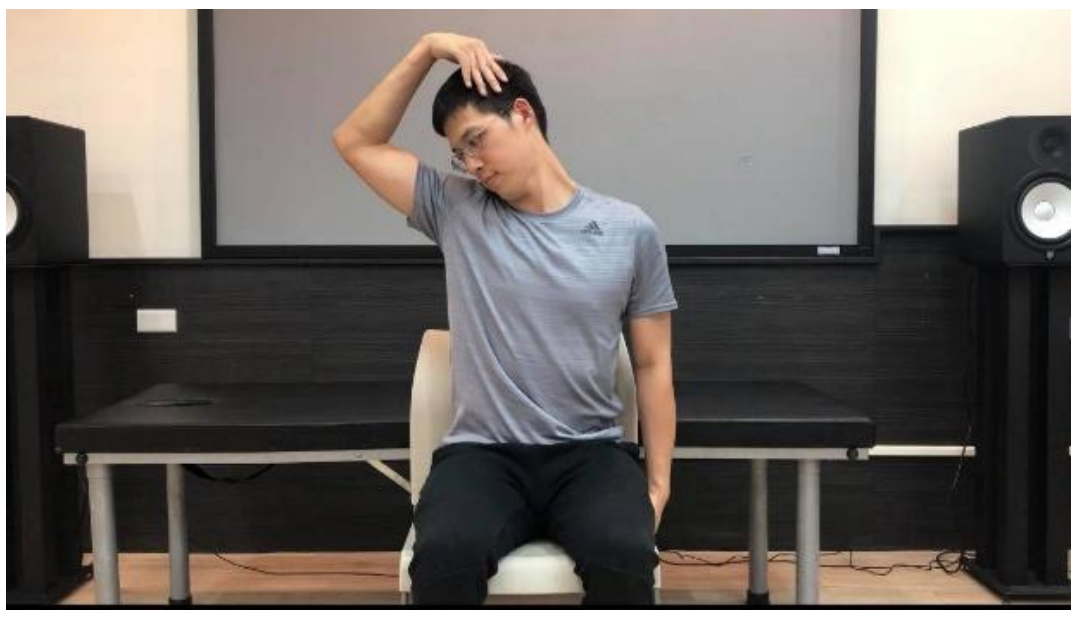

4. 前鋸肌強化運動：

A. 躺姿，患側手臂向上舉到約 90 度。

B. 手向天花板的方向碰及延伸，此時肩胛骨會離開床面。

C. 用力 3～5 秒後放鬆，再重複，至少做 10 次。

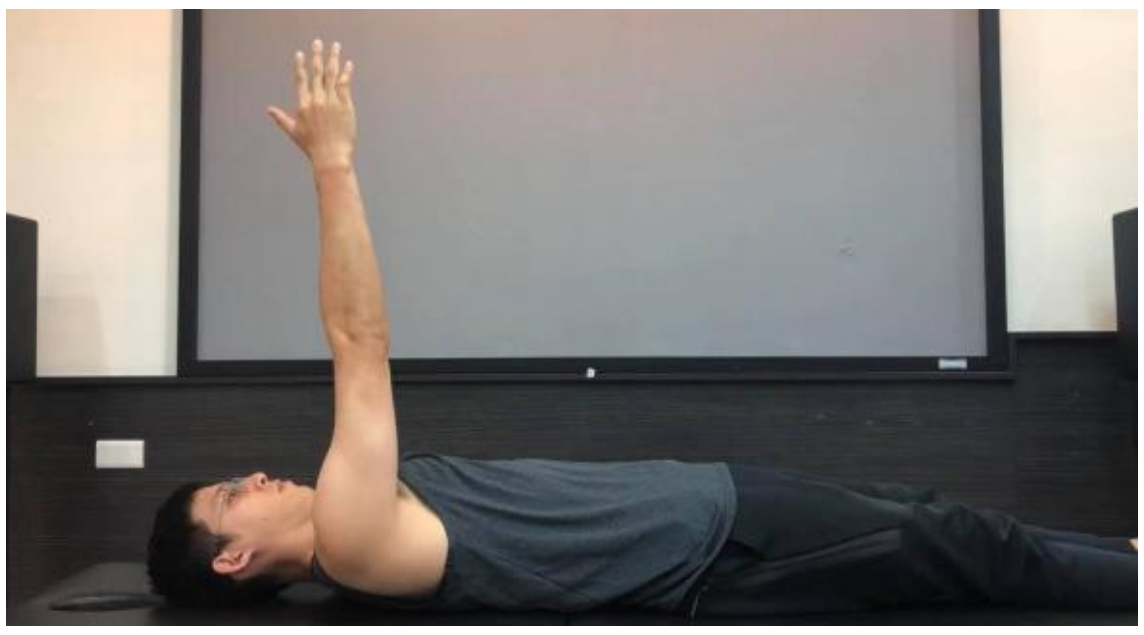

5. 中下斜方肌運動：

   A. 雙手先比出讚的姿勢後，大拇指朝外。

   B. 做出擴胸的動作，此時兩側肩胛骨往內夾。

   C. 夾住 3～5 秒後放鬆，再重複，至少做 10 次。

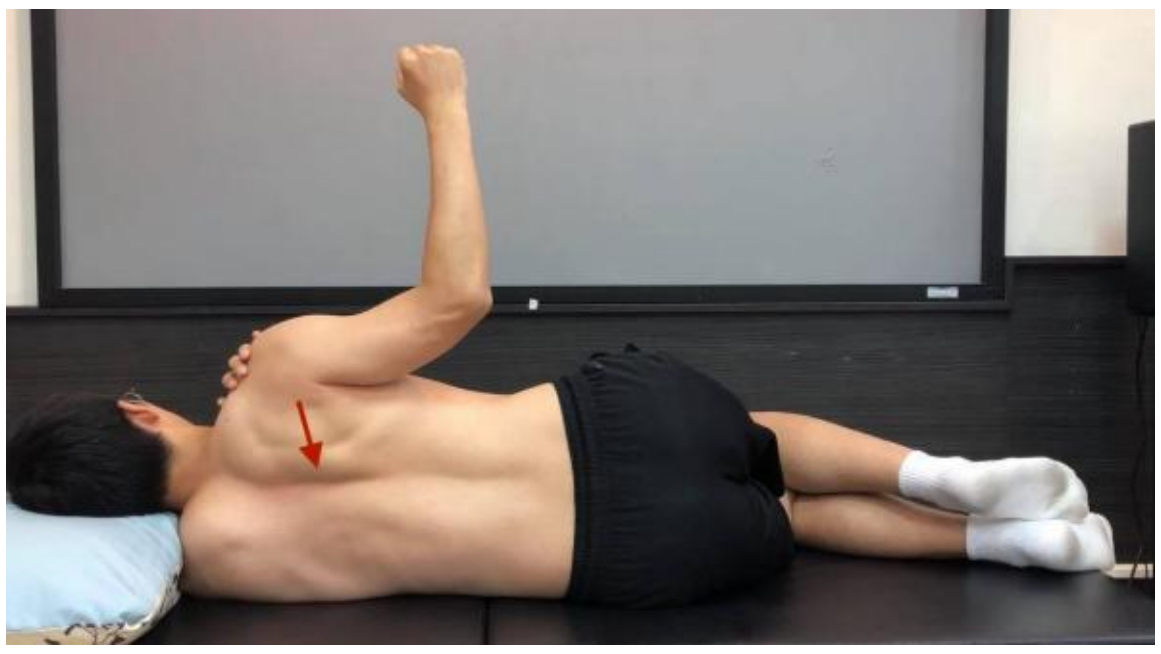

6. 推牆運動：

   A. 站姿不駝背，距離牆約一個手臂長。

   B. 雙手舉到胸前，寬度約肩寬再多一些。

   C. 類似伏地挺身動作，胸部及下巴往牆的方向靠但不碰到，此時肩胛骨內夾， 之後手肘再伸直回到起始位置再重複。

   D. 至少做 10 次

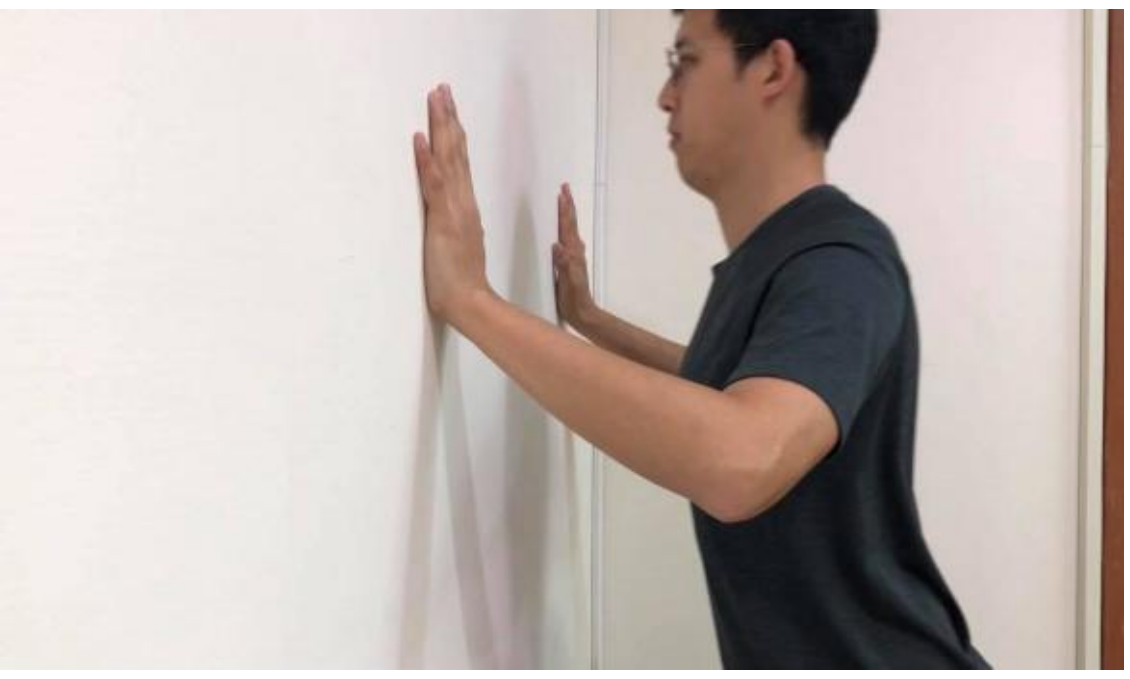

7. 手臂抬高伸展運動：

   A. 雙手握住雨傘或長棍。

   B. 患側手臂抬高，抬高到肩膀有緊的感覺再多一些，維持15～30 秒，再重複，至少做 5 次。

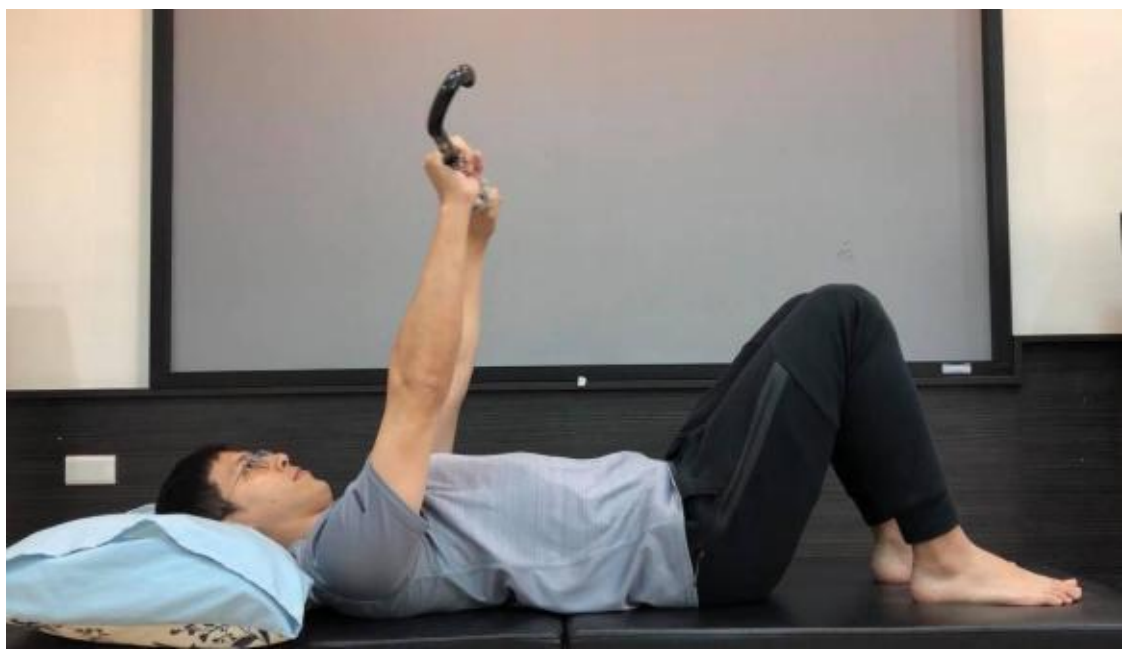

8. 手臂側邊抬高伸展：

   A. 雙手握住雨傘或長棍，患側手掌先朝前比出讚。

   B. 患側手臂抬高，抬高到肩膀有緊的感覺再多一些，維持15～30 秒，再重複，注意不聳肩，至少做 5 次。

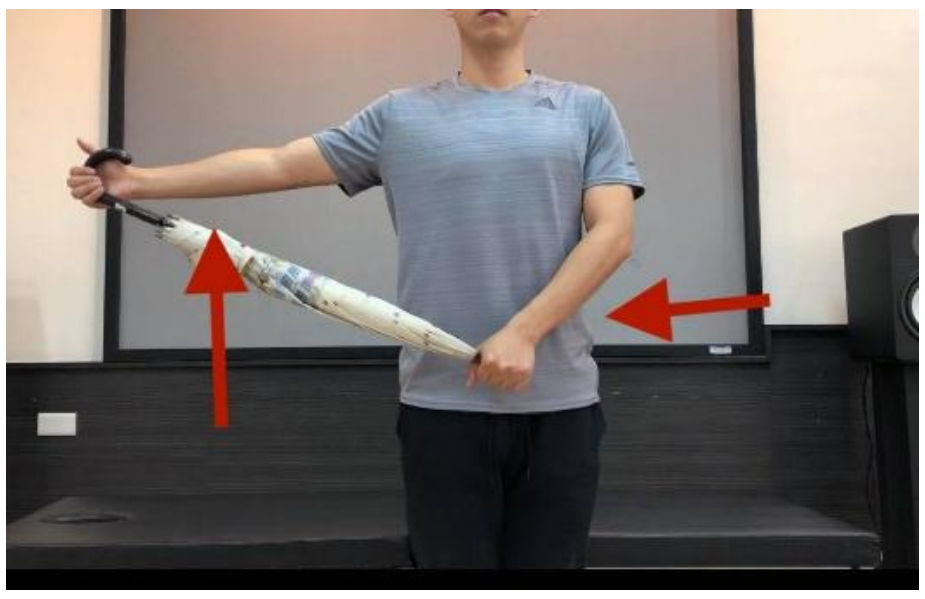

9. 手臂外轉伸展：

A. 雙手握住雨傘或長棍。

B. 患側手臂彎曲 90 度，上臂夾著毛巾。

C. 患側手臂做出類似開門動作，開到肩膀有緊的感覺再多一些，維持 15～30 秒，至少做 5 次。

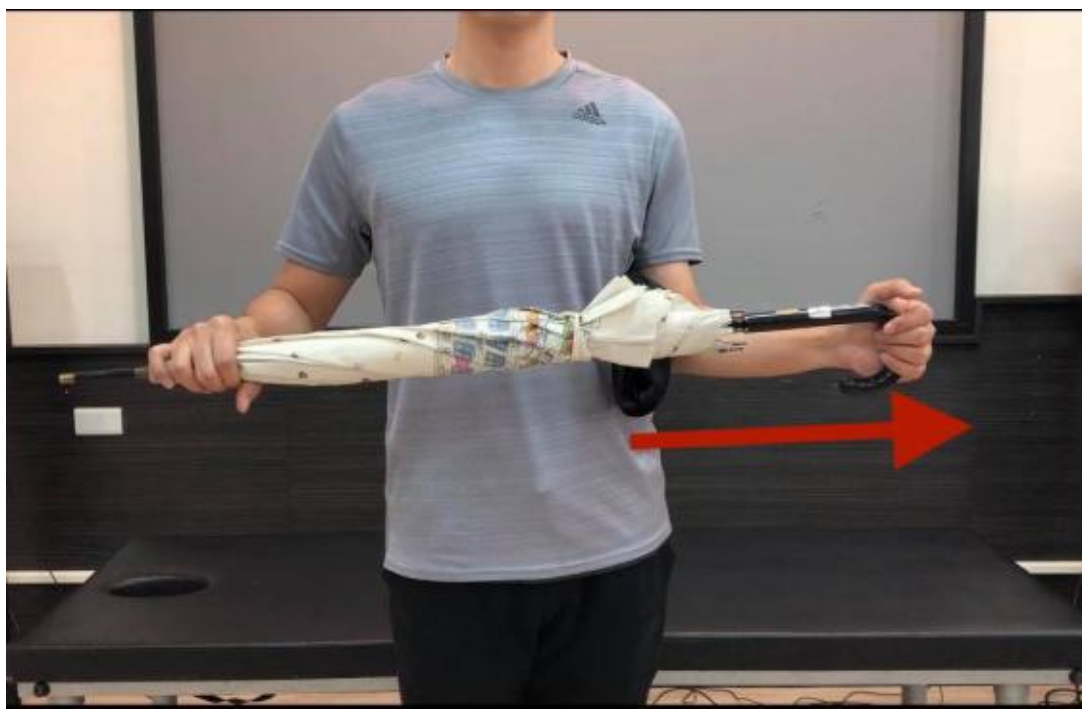

10. 摸被伸展運動：

A. 雙手握住雨傘或長棍。

B. 患側手在下，好側手在上。

C. 患側手臂向上摸背，摸到肩膀有緊的感覺再多一些，維持 15～30 秒，好手可以幫忙向上拉，至少做 5 次。

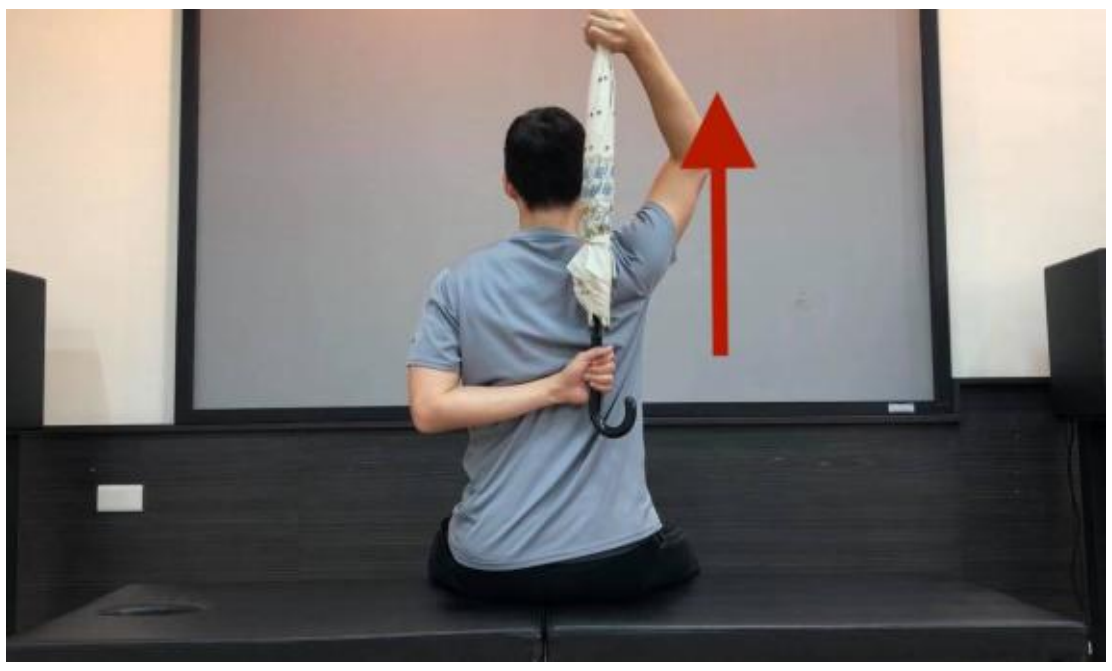

Supplement: Supplementary file 4 [file rehab-v13-e107314-s004.pdf]
