# Supplementary material for: Development, Usability, and Feasibility of a Pain-Stratified Smartphone-Based Home Intervention System for Patients With Adhesive Capsulitis: Exploratory Feasibility and Usability Trial
Source: JMIR Rehabil Assist Technol. 2026 Nov 5;13:e107314. doi: 10.2196/107314 (PMC13739270; doi:10.2196/107314)
Supplement: Supplementary file 5 [file rehab-v13-e107314-s005.pdf]

CONSORT 2010 checklist of information to include when reporting a pilot or feasibility trial*

Section/Topic	Item No	Checklist item	Reported on page No
**Title and abstract**			
	1a	Identification as a pilot or feasibility randomised trial in the title	Page 1, lines 1–3
	1b	Structured summary of pilot trial design, methods, results, and conclusions (for specific guidance see CONSORT abstract extension for pilot trials)	Pages 1–2, lines 4–52
**Introduction**			
Background and objectives	2a	Scientific background and explanation of rationale for future definitive trial, and reasons for randomised pilot trial	Pages 2–4, lines 54–131
	2b	Specific objectives or research questions for pilot trial	Page 4, lines 116–131
**Methods**			
Trial design	3a	Description of pilot trial design (such as parallel, factorial) including allocation ratio	Page 8, lines 247–254, 288–289
	3b	Important changes to methods after pilot trial commencement (such as eligibility criteria), with reasons	age 8, lines 263–264
Participants	4a	Eligibility criteria for participants	Page 8, lines 266–271
	4b	Settings and locations where the data were collected	Page 8, lines 254–257; page 12, lines 405–406
	4c	How participants were identified and consented	Pages 8–9, lines 255–257, 287–288
Interventions	5	The interventions for each group with sufficient details to allow replication, including how and when they were actually administered	Pages 4–7, lines 134–241; pages 8–

			10, lines 279–285, 305–329
Outcomes	6a	Completely defined prespecified assessments or measurements to address each pilot trial objective specified in 2b, including how and when they were assessed	Pages 10–12, lines 331–347, 366–389
	6b	Any changes to pilot trial assessments or measurements after the pilot trial commenced, with reasons	Page 12, lines 389–391
	6c	If applicable, prespecified criteria used to judge whether, or how, to proceed with future definitive trial	Pages 10–11, lines 348–364
Sample size	7a	Rationale for numbers in the pilot trial	Page 8, lines 271–277
	7b	When applicable, explanation of any interim analyses and stopping guidelines	Not applicable
Randomisation:			
Sequence generation	8a	Method used to generate the random allocation sequence	Page 9, lines 288–293
	8b	Type of randomisation(s); details of any restriction (such as blocking and block size)	Page 9, lines 288–291; page 12, lines 411–413
Allocation concealment mechanism	9	Mechanism used to implement the random allocation sequence (such as sequentially numbered containers), describing any steps taken to conceal the sequence until interventions were assigned	Page 9, lines 289–293; page 12, lines 411–413
Implementation	10	Who generated the random allocation sequence, who enrolled participants, and who assigned participants to interventions	Page 9, lines 287–293
Blinding	11a	If done, who was blinded after assignment to interventions (for example, participants, care providers, those assessing outcomes) and how	Page 9, lines 294–297
	11b	If relevant, description of the similarity of interventions	Page 10, lines 317–323
Statistical methods	12	Methods used to address each pilot trial objective whether qualitative or quantitative	Page 12, lines 392–401
**Results**			
Participant flow (a	13a	For each group, the numbers of participants who were approached and/or assessed for eligibility, randomly	Pages 12–13,

diagram is strongly recommended)		assigned, received intended treatment, and were assessed for each objective	lines 404–413; Figure 2 (page 13, line 425)
	13b	For each group, losses and exclusions after randomisation, together with reasons	Page 12, lines 413–414; page 13, lines 429–430
Recruitment	14a	Dates defining the periods of recruitment and follow-up	Page 8, lines 254–255
	14b	Why the pilot trial ended or was stopped	Page 12, lines 414–416
Baseline data	15	A table showing baseline demographic and clinical characteristics for each group	Pages 12–13, lines 417–423; Table 1 (page 13, line 426)
Numbers analysed	16	For each objective, number of participants (denominator) included in each analysis. If relevant, these numbers should be by randomised group	Page 13, Figure 2; Tables 2–4 (pages 14–16)
Outcomes and estimation	17	For each objective, results including expressions of uncertainty (such as 95% confidence interval) for any estimates. If relevant, these results should be by randomised group	Pages 13–17, lines 429–491; Tables 2–5; Figures 3–4 (pages 17–18)
Ancillary analyses	18	Results of any other analyses performed that could be used to inform the future definitive trial	Pages 22–23, lines 646–688
Harms	19	All important harms or unintended effects in each group (for specific guidance see CONSORT for harms)	Page 12, lines 416–417; page 13, lines

			429–431
	19a	If relevant, other important unintended consequences	Not applicable
**Discussion**			
Limitations	20	Pilot trial limitations, addressing sources of potential bias and remaining uncertainty about feasibility	Pages 23–24, lines 689–729
Generalisability	21	Generalisability (applicability) of pilot trial methods and findings to future definitive trial and other studies	Pages 22–24, lines 646–688, 690–694, 725–729
Interpretation	22	Interpretation consistent with pilot trial objectives and findings, balancing potential benefits and harms, and considering other relevant evidence	Pages 18–22, lines 497–523, 543–645; pages 24–25, lines 730–754
	22a	Implications for progression from pilot to future definitive trial, including any proposed amendments	Pages 22–23, lines 646–688; page 25, lines 751–754
**Other information**			
Registration	23	Registration number for pilot trial and name of trial registry	Not registered; explained on pages 1–2, 8, 24 (lines 46–50, 259–263, 713–725)
Protocol	24	Where the pilot trial protocol can be accessed, if available	Pages 25, lines 756–757
Funding	25	Sources of funding and other support (such as supply of drugs), role of funders	Page 25, lines 758–762
	26	Ethical approval or approval by research review committee, confirmed with reference number	Page 8, lines 257–259

Citation: Eldridge SM, Chan CL, Campbell MJ, Bond CM, Hopewell S, Thabane L, et al. CONSORT 2010 statement: extension to randomised pilot and feasibility trials. BMJ. 2016;355. This is an Open Access article distributed in accordance with the terms of the Creative Commons Attribution (CC BY 3.0) license (http://creativecommons.org/licenses/by/3.0/), which permits others to distribute, remix, adapt and build upon this work, for commercial use, provided the original work is properly cited.

*We strongly recommend reading this statement in conjunction with the CONSORT 2010, extension to randomised pilot and feasibility trials, Explanation and Elaboration for important clarifications on all the items. If relevant, we also recommend reading CONSORT extensions for cluster randomised trials, non-inferiority and equivalence trials, non-pharmacological treatments, herbal interventions, and pragmatic trials. Additional extensions are forthcoming: for those and for up-to-date references relevant to this checklist, see www.consort-statement.org.
